# Comprehensive Characterization of Cytochrome P450s Reveals Candidate Enzymes Involved in the Metabolic Fate of Absorbed Volatile Organic Compounds in Potato

**DOI:** 10.3390/antiox15091107

**Published:** 2026-09-02

**Authors:** Milica D. Bogdanović, Nina Devrnja, Katarina B. Ćuković Janićijević, Sofija Stupar, Slađana I. Todorović, Jelena Savić

**Affiliations:** Department for Plant Physiology, Institute for Biological Research “Siniša Stanković”—National Institute of the Republic of Serbia, University of Belgrade, 11108 Belgrade, Serbia; milica.bogdanovic@ibiss.bg.ac.rs (M.D.B.); nina.devrnja@ibiss.bg.ac.rs (N.D.); katarina.cukovic@ibiss.bg.ac.rs (K.B.Ć.J.); sofija.stupar@ibiss.bg.ac.rs (S.S.); slatod@ibiss.bg.ac.rs (S.I.T.)

**Keywords:** plant–plant interactions, essential oil, oxidative detoxification, *CYP81D* genes, terpene metabolism, potato (*Solanum tuberosum* L.), French marigold (*Tagetes patula* L.), genome-wide bioinformatics characterization

## Abstract

Plants are continuously exposed to volatile organic compounds (VOCs) emitted by neighbors. Although the mechanisms governing VOC uptake and metabolism remain unclear, cytochrome P450 monooxygenases (CYP450s) are thought to participate in the detoxification and metabolic conversion of absorbed VOCs. Data from previously conducted cDNA microarray transcriptomic profiling in potato exposed to French marigold essential oil (FM-EO) was here used to filter differentially expressed sequences, and identified 54 unique CYP450 transcripts. Among the 10 most highly expressed sequences, two *CYP81D1*-like (*81D1-1* and *81D1-2*) and one *CYP81D11*-like (*81D11-1*) transcripts were found. RT-qPCR confirmed their strong induction within 8 h of volatile exposure. Comprehensive bioinformatics identified the most highly induced *81D1-1* gene as a CYP450 containing a predicted N-terminal hydrophobic signal or membrane-anchor region, the conserved heme-binding signature motif, and regulatory elements associated with oxidative stress responses. The other *81D11-1* gene, exhibiting a comparable expression level, was annotated only as a heme-binding protein but possessed seven distinct cis-regulatory elements, suggesting high transcriptional plasticity. Machine learning predictions assigned the highest interaction probability to (Z)-β-ocimene, whereas structure-based docking yielded the most favorable mean score for piperitone. This study provides the first characterization of the potato CYP450 superfamily in the context of volatile-mediated plant–plant interactions and identifies two CYP81D members as strong candidates for the oxidative metabolism of absorbed VOCs. The results support a proposed detoxification pathway in which CYP81-mediated oxidation precedes glutathione conjugation and intracellular sequestration of VOCs. These candidate genes provide a valuable foundation for future functional studies and may facilitate the development of sustainable crop protection strategies based on volatile-mediated plant defense.

## 1. Introduction

For plants as sessile organisms, an environment can provide stressful conditions, making rapid “communication” and timely responses to stress signals essential for survival. When a receiver plant detects volatile organic compounds (VOCs) emitted by neighbors, either as constitutively released essential oils (EOs) or specific blends emitted upon stress situations, it perceives them as airborne information signals and translates them into internal biochemical cascades [1]. In recent years, the potential of VOCs to influence plant physiology, particularly by modulating defense responses, has been increasingly recognized as a promising approach for enhancing plant immunity and stress tolerance in sustainable agriculture [2]. VOCs (primarily monoterpenes, sesquiterpenes, and phenylpropanoids) are highly lipophilic, with low molecular weight and high volatility [3]. While, in the producing plant, VOCs serve as essential secondary metabolites that contribute to its ecological adaptation and survival, the receiving plant is likely to perceive them as foreign chemicals capable of interfering with cellular homeostasis or signaling. Consequently, it may recruit the same metabolic machinery that is commonly involved in the detoxification and metabolism of exogenous synthetic chemicals as xenobiotics [3,4,5]. The fate of these chemically reactive compounds [6] within the tissues of receiver plants is not yet fully understood. VOCs enter the intercellular spaces of leaves either by adsorption on the leaf surface or through the stomata, where they are processed through reactions such as oxidation, reduction, glutathionylation, and glycosylation [7]. Through these processes potentially harmful volatiles are redirected into the vacuole or the apoplast, but also, plants can further process, reemit, or recycle sequestered conjugates by incorporating them into macromolecules like phytohormones, lignin, and cellulose [4]. The whole metabolic turnover of up-taken VOCs consists of three phases: (I) modification or functionalization, (II) conjugation, and (III) sequestration through cellular compartmentalization. Despite being less extensively investigated, the modification phase represents a critical step that directs the subsequent metabolic fate of xenobiotics. This phase involves the introduction or unmasking of reactive and polar functional groups within xenobiotic molecules, predominantly mediated by cytochrome P450 monooxygenases and, to a lesser extent, various esterases and hydrolases [4,8].

A growing body of research confirms that cytochrome P450 enzymes (CYP450s) play a pivotal role in the detoxification of potentially harmful compounds up-taken or generated during plant stress responses, by catalyzing their oxidative transformation into more polar metabolites suitable for further detoxification and sequestration [3,8,9,10,11]. In general, CYP450s catalyze key reactions in the majority of plant-specific metabolic pathways, including many rate-limiting steps, and are encoded by approximately 1% of plant protein-coding genes [12,13]. Numerous studies have demonstrated that CYP450 genes are highly inducible under stress conditions and exhibit strong tissue-specific expression patterns, with particularly high expression levels in leaves and epidermal tissues [14,15].

Cytochrome P450s constitute a highly diverse superfamily in which enzymes are classified into clans, families, and subfamilies based on amino acid sequence identity [14]. CYP450 enzymes contain a heme prosthetic group that enables oxidative transformations of substrates [5,16]. These reactions are often accompanied by the production of reactive oxygen species (ROS) and oxidized intermediates that must subsequently be managed by cellular detoxification systems. Plant CYP450s exhibit considerable substrate promiscuity, enabling them to metabolize a wide range of structurally diverse endogenous and exogenous compounds [17]. In addition to classical oxygenation reactions, CYP450s catalyze diverse transformations including dealkylation, desaturation, isomerization, and carbon–carbon bond cleavage, reflecting remarkable catalytic plasticity that likely arose through extensive gene duplication and functional diversification [11]. This versatility is particularly evident in the detoxification of xenobiotics, as numerous herbicide-metabolizing CYP450s have been identified across crop species [18,19]. Among them, members of the CYP81 family are increasingly recognized for their ability to metabolize a broad spectrum of chemically diverse herbicides and xenobiotics [20,21,22,23]. CYP81 family members serve two primary roles in the context of detoxification: the direct metabolic detoxification of diverse xenobiotics, and the integration of stress signaling pathways triggered by harmful chemicals. It seems that different members of the CYP81 family have diverged in their roles, some specialized in the direct detoxification, while others having indirect supportive roles in managing xenobiotic-induced stress, as components of stress signaling pathways [9,10,13].

The broad catalytic capacity of CYP450s suggests that they may play an important role in the metabolism of different exogenous plant-derived volatiles perceived during plant–plant communication. Although numerous studies have demonstrated that VOCs can induce defense-related responses in neighboring plants [3], the molecular mechanisms underlying their metabolic processing after uptake remain poorly understood. In particular, it is unclear whether exposure to volatile signals activates xenobiotic-like detoxification pathways involving CYP450 enzymes, and to what extent. A previous study revealed a strong induction in detoxification-related glutathione-S-transferase (*GST*) transcript accumulation in potato exposed to volatiles of French marigold EO [24]. The study demonstrated that exposure to EO volatiles modulated glutathione (GSH) redox cycling during the early response (up to 8 h) and provided evidence of the induced burst of ROS. It was previously shown that dicots rely on CYP450s, mostly CYP72 enzymes, which, together with GSTs, are involved in detoxifying herbicides [25]. However, the relationship between EO-induced ROS burst, GSH redox cycling, and the pronounced induction of *GST* gene expression remains to be elucidated in detail to provide valuable insights into the metabolic fate of absorbed volatile compounds in plant cells.

Potato (*Solanum tuberosum* L.) possesses a large and diverse CYP450 repertoire [26,27,28], providing a suitable model for investigating CYP450-mediated responses to volatile exposure. Two genomic sequences and annotations are available for potato: NCBI hosts a version of potato genome SolTub_3.0 published in 2011 (*S. tuberosum* strain: DM 1-3 516 R44) (https://www.ncbi.nlm.nih.gov/datasets/genome/GCF_000226075.1; accessed on 5 June 2024), while the Spud DB Potato Genomics Resource hosts an improved long-read chromosome-scale genome assembly v6.1 (*S. tuberosum* Group Phureja DM 1-3 516 R44) (https://spuddb.uga.edu/dm_v6_1_download.shtml; accessed on 20 January 2026) from 2020. Depending on the genome version used and the conditions for CYP450 filtering, the total number of potato *CYP450* genes was estimated to be 488 [27], 558 [28], or 563 [26]. Potato CYP450 proteins are classified into nine clans and 46 [26], 44 [28], or 41 families [27]. They are broadly divided into A-type (CYP71 clan) and non-A type (CYP51, CYP72, CYP74, CYP85, CYP86, CYP97, CYP710, and CYP711 clan) [26,27,28]. Like in other plants, potato *CYP450* genes are described as vital regulators of plant growth, development, hormone signaling, and adaptation to various biotic and abiotic stresses [26,27,28]. A large proportion of *CYP450* genes in potato are transcriptionally active across various tissues and environmental conditions. Among the *CYP450* genes studied by RNA-seq, some are highly expressed in almost all potato tissues, indicating their functional importance throughout the potato lifecycle, while others showed distinct differential expression across different potato organs, indicating more specialized roles [26]. Some *CYP450* genes in potato also proved important and upregulated in stress responses, like in response to *Phytophthora infestans* (late blight) [27], salt stress [26], and osmotic or heavy metal stress [28].

The present study aimed to characterize CYP450-associated transcriptomic responses in potato plants exposed to volatiles of French marigold essential oil (FM-EO) and to explore the structural and functional features of the most responsive CYP450 members using in silico approaches. French marigolds are widely recognized for their insecticidal and insect repellent properties and are frequently utilized in companion planting systems. However, increasing evidence suggests that volatile compounds also influence neighboring potato plants [24,29,30]. The findings of this study will contribute to a wide investigation of the effects of FM-EO on potato which will provide new insights into the molecular basis of VOC-mediated plant–plant interactions. Elucidating the consequences of these interactions for the recipient plant and evaluating the potential of intercropping these two species as a sustainable agroecological strategy are of considerable importance. By integrating transcriptomic and computational analyses, this study sought to identify candidate CYP450s involved in VOC metabolism and detoxification. To our knowledge, this represents the first integrative investigation of CYP450-associated responses in potato following exposure to EO volatiles.

## 2. Materials and Methods

### 2.1. Analyses of CYP450 Expression in Potato Exposed to French Marigold Essential Oil

The exposure of 3-week-old potato plants (*Solanum tuberosum* L.) to the French marigold (*Tagetes patula* L.) essential oil (FM-EO) was described in Stupar et al. [30]. Briefly, potato was exposed to FM-EO for 2, 4, 6, or 8 h to evaluate how exposure duration influenced the dynamics of plant responses. Leaf samples were collected from a middle section of three to four representative plants per experimental group or corresponding controls as biological replicates (*n* = 3 or 4). Liquid-frozen material was used for RT-qPCR and Western blot analyses.

#### 2.1.1. Filtering and Annotation of CYP450 Sequences from cDNA Microarray Data of Potato Exposed to FM-EO

Potato plants exposed to FM-EO for 8 h, along with their corresponding control plants, were previously subjected to cDNA microarray analysis [30]. Briefly, raw data were extracted using Agilent Feature Extraction Software (v11.0.1.1) and selected gProcessedSignal values were transformed by logarithm and normalized by the quantile method. Statistical significance of the cDNA microarray expression data was determined using fold change (fc) and independent *t*-tests in which the null hypothesis was that no difference exists among 2 groups. To account for multiple comparison testing across microarray probes, raw *p*-values were adjusted using the Benjamini–Hochberg False Discovery Rate (FDR) procedure. The list of differentially expressed (DE) probes with |fc| ≥ 2 cut-off and adjusted bh.*p*val < 0.05 (*n* = 4) was used for further filtering of CYP450 sequences.

CYP450 sequences were identified by keyword (P450) search in the Description column. Found sequences were further annotated and classified using NCBI and InterPro search, via Primary Accession identifier. CYP450 probes were sorted by fc, and the top ten probes were selected for detailed annotation, classification, and protein domain information extraction using the same services. Out of those (all belonging to CYP72-like and 81-like families), 4 different transcripts were chosen for gene expression dynamic studies (XM_006339781.1; XM_006362014.2; XM_015305409.1; XM_006348243.2).

#### 2.1.2. RT-qPCR Analysis of CYP450 Genes Expression in Potato Plants Exposed to FM-EO

Total RNA was isolated from 100 mg of frozen, powdered leaf tissue following a protocol based on Gasic et al. [31]. The quantity and purity of the isolated RNA were determined spectrophotometrically using a NanoPhotometer^®^ N60 (Implen GmbH, Munich, Germany). Total RNA was treated with DNase I and reverse transcribed (RT) into complementary cDNA using oligo d(T)_16_ primers, following the manufacturer’s instructions.

Quantitative real-time PCR (qPCR) analyses were performed on the same way as previously described by the authors [24] using SYBR Green chemistry. Each reaction contained 25 ng of cDNA. All reactions were conducted in biological triplicates (*n* = 3). Gene-specific primers of four *CYP450* sequences, previously chosen for gene expression studies, were designed using Primer-BLAST (NCBI; https://www.ncbi.nlm.nih.gov/tools/primer-blast/ (accessed on 29 August 2026)); Table S1). Primer specificity was confirmed by BLAST analysis, electrophoretic separation of RT-PCR products on 1.2% agarose gels, and melting curve analysis following qPCR amplification.

Gene expression levels were normalized to the internal reference gene *18S rRNA* and calculated relative to the corresponding non-exposed control using the 2^−ΔΔCt^ method [32], and presented as the fold change (FC) of the gene expression level. Results were presented as log_2_-transformed fold changes (log_2_FC). Statistical comparisons between each experimental group and its respective control were performed using Student’s *t*-test, with predefined statistical thresholds set at *p* ≤ 0.05 (*) and *p* ≤ 0.10 (**). Differences among treatments were evaluated using one-way ANOVA followed by Fisher’s least significant difference (LSD) test at *p* ≤ 0.05, and statistically distinct means are indicated by different letters (*n* = 3).

#### 2.1.3. Immunoblotting Detection of CYP81 Proteins in Potato Plants Exposed to FM-EO

Total proteins were extracted from potato leaves exposed to FM-EO for 8 h and corresponding controls. Plant material was ground to a fine powder in liquid nitrogen in the presence of ice-cold 50 mM Tris-HCl buffer (pH 7.6) containing 10 mM ethylenediaminetetraacetic acid (EDTA, pH 8.0), 1 mM dithiothreitol (DTT), 1 mM phenylmethylsulfonyl fluoride (PMSF), 10% (*v*/*v*) glycerol, and 5% (*w*/*v*) insoluble polyvinylpolypyrrolidone (PVPP), at a 2:1 (*v*/*w*) buffer-to-tissue ratio. The homogenate was centrifuged at 12,000× *g* for 10 min at 4 °C, and the supernatant was collected for further analyses. Total soluble protein concentration was determined using the Bradford method [33] with bovine serum albumin (BSA) as a standard. For further Western blot analysis, protein extracts from three biological replicates per sample were pooled in equal amounts of total proteins.

Protein extracts were separated by sodium dodecyl sulfate–polyacrylamide gel electrophoresis (SDS-PAGE), using the Mini-Protein II system (Bio-Rad, Hercules, CA, USA). Equal amounts of proteins (10 μg per sample) were loaded onto 10% resolving gels with 4% stacking gels. Electrophoresis was performed at 120 V for 90 min at room temperature. Proteins were transferred onto PVDF membranes (0.2 μm pore size; Bio-Rad, USA) using a Mini Trans-Blot module (Bio-Rad, USA) at 100 V for 70 min at 4 °C. Membranes were blocked overnight at 4 °C in 10% (*w*/*v*) non-fat dry milk in phosphate-buffered saline (PBS) containing Tween 20 (T-PBS). Blots were then incubated for 2 h at room temperature with rabbit cytochrome P450 81 primary antibody (Anti-CYP81; Agrisera, Vännäs, Sweden, AS20 4445) diluted 1:5000 in blocking solution. Membranes were washed three times for 10 min in T-PBS and incubated for 2 h at room temperature with goat anti-rabbit IgG-HRP secondary antibody (Agrisera, AS09 602) diluted 1:25,000. Protein bands were visualized using an enhanced chemiluminescence detection system. To verify equal protein loading, membranes were probed with rabbit Rubisco large subunit, form I antibody (Anti-RbcL, Agrisera, AS03 037) diluted 1:10,000, followed by goat anti-rabbit IgG-HRP secondary antibody (1:25,000), using the same procedure as described above.

### 2.2. In Silico Characterization of Potato CYP450 Family

The potato whole genome data, coding DNA sequences (CDSs), and protein sequences were obtained from the Spud DB Potato Genomics Resource (http://solanaceae.plantbiology.msu.edu) [34]. *CYP450* genes were identified using Functional Annotation Search and Pfam Accession Search (PF00067). The coordinates of exons, introns, and untranslated regions (UTRs) of potato *CYP450* genes were determined using the Gene Structure Display Server 2.0 (GSDS) software (http://gsds.gao-lab.org) [35].

InterPro search (https://www.ebi.ac.uk/interpro/search/sequence/ (accessed on 29 August 2026)) on CYP450 predicted protein sequences was used to identify classification of representative protein domains by the following annotation tools and databases within InterPro: Phobius, CDD, PANTHER, Pfam, FunFam, Gene3D, SignalP, PRINTS, ProSitePatterns, TMHMM, and Gene Ontology (GO) functional annotations. Additional GO term annotations were obtained with Blast2GO (https://www.blast2go.com/). Physical and chemical parameters were calculated using the ExPASY ProtParam tool (https://web.expasy.org/protparam/).

#### 2.2.1. Promoter Analysis of CYP81 Gene Family in Potato

Promoter sequences of *CYP81* potato genes (deposited in RADaR, https://radar.ibiss.bg.ac.rs/handle/123456789/8179 (accessed on 29 August 2026)) were extracted from the potato genome from the Spud DB database using the open-source software UGENE v53.1 [36]. Promoter regions were considered within a region from 1000 nucleotides upstream to 100 nucleotides downstream of the transcription start site. In the case of closely spaced genes, where the −1000 bp region overlapped with a neighboring gene or its 3′ end, the promoter region was limited to the available intergenic sequence between them. Putative cis-regulatory elements (CREs) in promoter regions were identified using the PlantCARE database (https://bioinformatics.psb.ugent.be/webtools/plantcare/html (accessed on 29 August 2026)) [37]. The identified motifs were subsequently classified according to their predicted regulatory functions using the Simple PlantCARE Classify tool from TBtools software v2.441 [38].

Families of transcription factors (TFs) capable of binding specific motifs in *CYP81* promoters were identified using PlantRegMap (http://plantregmap.gao-lab.org/binding_site_prediction.php (accessed on 29 August 2026)) [39]. The list of TFs was obtained from *Solanum tuberosum* L. The threshold value for the *p*-value parameter was set to 10^−5^, and only TF-binding sites with *q*-values lower than 0.05 were selected for further analysis, according to [40]. TF-binding sites and TF families were visualized using TBtools software. Gene Ontology (GO) annotations were assigned to the identified TFs using information retrieved from the plant TF database PlantTFDB (v5.0, http://planttfdb.gao-lab.org/) [41], in order to describe the biological processes in which they are involved.

#### 2.2.2. Three-Dimensional Structures, Conserved Domains, and Cellular Localization of CYP81 Proteins of Potato

The proposed three-dimensional (3D) tertiary structures of CYP81 potato proteins were predicted using AlphaFold [42,43] and visualized with UCSF ChimeraX (version 1.11.1) [44]. Model confidence was evaluated based on predicted local distance difference Test (pLDDT) scores, and the structures were colored in shades from dark blue (high prediction confidence) to red (potentially disordered regions).

The heme-binding domains were identified in the amino acid sequences of potato CYP81 proteins by scanning for the conserved signature motif (FxxGxxxCxG) using the ScanProsite tool (https://prosite.expasy.org) [45]. The positions of signal peptides and transmembrane domains were predicted by InterPro Phobius service (https://www.ebi.ac.uk/interpro/search/sequence/ (accessed on 29 August 2026)). Based on the obtained coordinates, the major protein domains were graphically represented using IBS 1.0 software (http://ibs.biocuckoo.org) [46] through the Domain Graph (DOG) package [47]. Transmembrane, intra- and extracellular protein domains predicted by Phobius were visualized using Protter (http://wlab.ethz.ch/protter/# (accessed on 29 August 2026)).

#### 2.2.3. Protein–Protein Interactions of CYP81s in Potato

The protein–protein interaction networks for CYP81 proteins of potato were predicted using STRING (https://string-db.org) [48], with Arabidopsis as a reference species, and adding two levels of nodes to enable identification of functional interactions as well as GO enrichment related to molecular function.

### 2.3. Enzyme-Substrate Prediction and Docking Analysis of Potato CYP81 Enzymes and French Marigold Essential Oil Constituents

To estimate enzyme–substrate interaction, protein sequences of the CYP81 family were compared to eight constituents of FM-EO [30]: terpinolene, limonene, (*E*)-caryophyllene, and (*Z*)-β-ocimene, identified as the most abundant compounds in the essential oil, and piperitone, (*Z*)-tagetone, (*E*)-tagetone, and dihydrotagetone that were identified as promising structural targets of cytochrome P450 enzymes. These components together made up 84.1% of the total FM-EO. Protein sequences of proposed CYP450 members extracted from potato genome (Spud DB) and SMILE structures of chosen compounds, retrieved from PubChem, were uploaded to Enzyme-Substrate Prediction (ESP) section of the P450Rdb v2.0 website (https://www.cellknowledge.com.cn/p450rdb_v2/prediction.html (accessed on 29 August 2026)) and analyzed by deep learning using ESM + GNN + XGBoost models to predict interaction probability, based on experimental evidence and biological pattern recognition [49]. Interaction probability was reported as a score between 0 and 1, where scores closer to 1 indicated higher interaction probability.

The interaction of chosen essential oil constituents and CYP81 candidates was further explored by preliminary in silico docking to evaluate potential binding interactions using the CB-Dock2 tool (http://183.56.231.194:8001/cb-dock2/php/blinddock.php (accessed on 29 August 2026)) [50]. The proposed 3D structure of CYP81s was previously obtained using AlphaFold. Each structure was aligned based on sequence similarity to the known CYP450 X-ray crystal plant protein structures (PDB IDs: 7x2q, 8e83, 6A15, 6l8h) from multiple databases, including the Protein Data Bank (PDB), the AlphaFold Database clustered to 50% sequence identity (AFDB50), AllTheBacteria (ATBC), the Big Fantastic Virus Database (BFVD), Viro3D, and Kinetoplastid Parasites using Foldseek. The most similar CYP450 was used to transfer and align the heme ligand group, to better visualize docking positions. To verify the favorable position of heme, the distance between the Fe ion in the center of heme and the sulfur (SG) atom in cysteine (Cys) residue inside the heme-binding domain was measured in ChimeraX.

Protein 3D structures with aligned heme group and ligand sequences in .sdf format, retrieved from PubChem, were used for cavity-detection guided blind docking at the CB-Dock2 website (http://183.56.231.194:8001/cb-dock2/php/blinddock.php (accessed on 29 August 2026)), using the AutoDock Vina (version 1.1.2) method. Binding affinities (Vina score) were calculated in kcal/mol, and docking poses were visualized using ChimeraX. The ligand positions closest to the heme group and with suitable binding energy were chosen for further analysis. For each CYP81–ligand pair, the distance between the Fe ion and the ligand molecule from the closest C atom, was calculated to assess catalytic relevance using ChimeraX.

## 3. Results

### 3.1. Expression of CYP450s in Potato Plants Exposed to FM-EO

#### 3.1.1. Identification of DE CYP450 Sequences in cDNA Microarray of Potato Exposed to FM-EO

cDNA microarray data obtained after exposing potato plants to FM-EO for 8 h were analyzed. Out of 62,976 databases-selected features (8 × 60 K), a total of 61,307 designable probes covered 99.44% of features. Of 36,037 microarray probes that were filtered by flag, logarithm, and normalization across the entire raw dataset, a total of 3561 sequences comprised a significantly differentially expressed (DE) set with |fc| ≥ 2 and bh.*p*val < 0.05 (https://radar.ibiss.bg.ac.rs/handle/123456789/8350 (accessed on 29 August 2026)).

**Figure 1:**
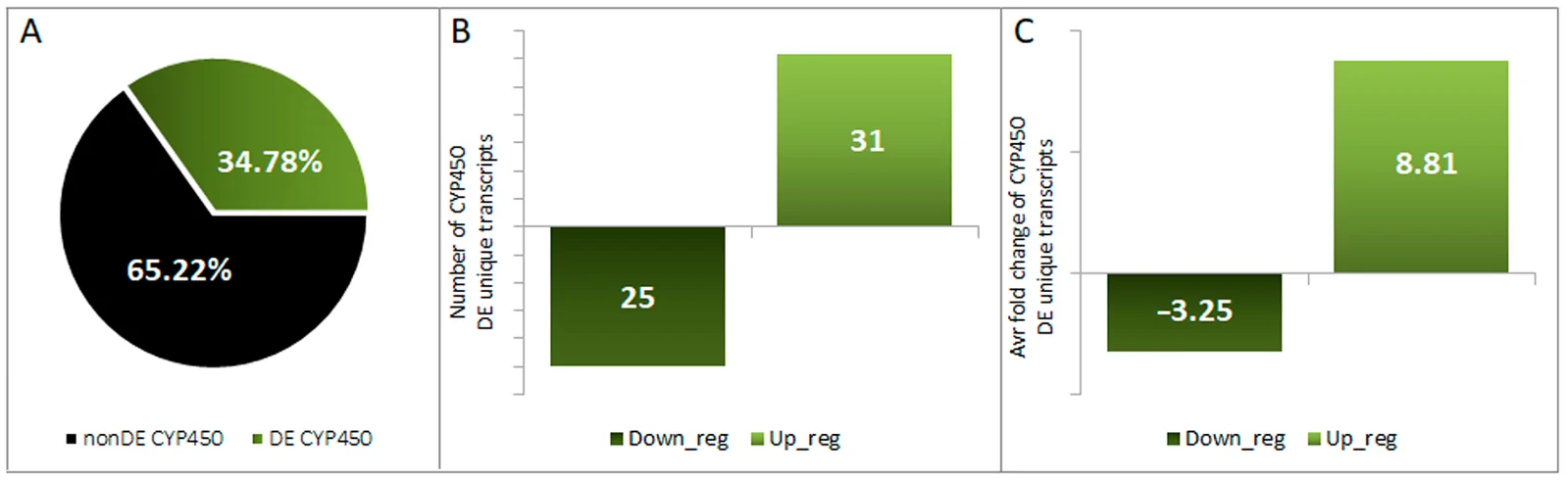
Differentially expressed (DE) CYP450 transcripts in cDNA microarray data of potato exposed to French marigold essential oil for 8 h. (**A**) Share of DE transcripts in all analyzed *CYP450* sequences. (**B**) Number of downregulated and upregulated DE transcripts, with (**C**) average values of fold change (fc) of these transcripts.

Among all filtered microarray probes subjected to analysis (36,037), *CYP450* transcripts were detected by 174 probes, of which 161 were unique transcripts determined by Primary Accession (Table S2). The subset of DE transcripts (3561) contained 59 probes corresponding to 54 unique *CYP450* transcripts. This makes 33.54% of all analyzed *CYP450* transcripts being DE in potato after exposure to FM-EO for 8 h (Figure 1A). Of all the DE transcripts, 29 were upregulated and 25 were downregulated (Figure 1B), with the average values of fc being −3.25 for downregulated ones and 9.90 for upregulated ones (Figure 1C).

The most DE probes and transcripts were identified next. Ten probes with the highest fc values belonged to eight different transcripts (Table 1). For two doubled probes, the mean was presented for transcript fc. All transcripts had annotations of plant cytochrome P450 monooxygenases, including representatives of three CYP450 subfamilies: 81D1-like, 81D11-like, and CYP72A219-like. In the CYP72A219-like group, three different genes were identified according to NCBI Primary Accessions, and one of them (XM_00634824x.2) was present with three transcript variants. The 81D1-like group was represented by two different genes, and the 81D11-like group by just one among the top ten highly expressed probes in microarray analysis.

For each of these highly-inducible CYP450 potato sequences, study-specific short labels are introduced and used consistently throughout the manuscript when referring to these sequences (Table 1).

#### 3.1.2. Classification of Potato CYP450s from cDNA Microarray of Potato Exposed to FM-EO

The 54 unique cDNA microarray transcripts, differentially expressed in potato exposed to FM-EO (Figure 1B), were classified in 23 CYP450 subfamilies, 52 of them belonging to E-class group I. The E-class group I designation specifies endoplasmic reticulum-bound, NADPH-reductase-dependent plant P450s belonging to the highly expanded CYP71 clan, which drives diverse specialized and secondary metabolic pathways. The most abundant subfamily in this class was CYP72A219-like, with 14 members (Table 2).

Classification according to the Conserved Domain Database (CDD), which uses a multi-tiered approach to organize CYP450 domains based on their evolutionary relationships and structural features, revealed the existence of 9 CDD classes (Table 3) that characterize 42 proteins coded from DE cDNA microarray-extracted sequences.

These sequences were also characterized in InterPro by conserved CYP450 cysteine heme–iron ligand signature (ProSitePatterns PS00086), signal peptide (SP), and transmembrane (TM), extracellular (EC) and cytoplasmic (CP) domains according to Phobius, and TMHMM transmembrane helix (Table 4).

When previous characterization was applied to the eight transcripts which were the most differently expressed potato CYP450 transcripts presented in Table 1, all were classified into the same E-class group I, and all had annotation of plant cytochrome P450 monooxygenases. Subclassification included two CDD classes—cd20653 (CYP81) and cd20642 (CYP72). In terms of predicted protein domains (Table 5), only two of those transcripts were characterized by signal peptide region (SP), while a transmembrane (TM) region was predicted in six out of eight transcripts (Phobius prediction). According to TMHMM prediction, four out of eight transcripts were characterized by the transmembrane helix structure.

#### 3.1.3. Expression Dynamic of CYP450 Genes in Potato Plants Exposed to FM-EO

Based on transcriptomic data obtained from cDNA microarray analysis, four genes out of the top eight with the highest fc were selected to monitor the expression dynamics following exposure to volatile compounds of FM-EO. Following primer design and validation, the genes from subfamilies 81D1-like (XM_006339781.1 labeled as *81D1-1*; XM_006362014.2 labeled as *81D1-2*) and 81D11-like (XM_015305409.1 labeled as *81D11-1*), together with one representative of the CYP72A219-like subfamily (XM_006348243.2 labeled as *CYP72-2*), all showing reliable and specific amplification, were chosen for RT-qPCR analysis.

**Figure 2:**
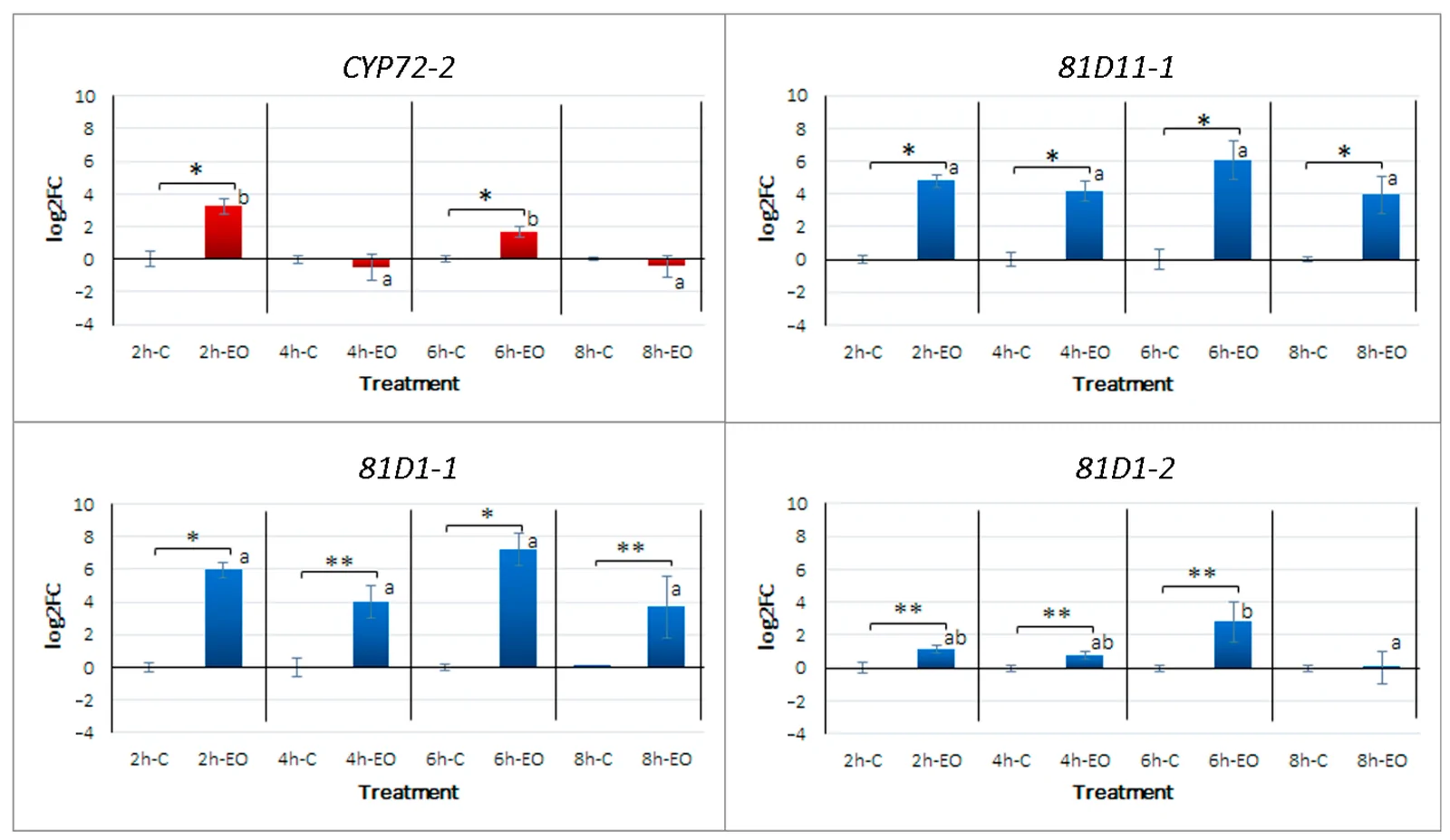
RT-qPCR expression profiles of selected *CYP450* genes (*CYP72-2, 81D1-1*, *81D1-2*, and *81D11-1*) in potato exposed to French marigold essential oil (EO) for different time periods (2, 4, 6, and 8 h). The fold change (FC) of the gene expression was obtained after ddCt normalization to the expression of the reference *18S* gene and to the expression in non-exposed controls (left bars; log_2_FC = 0) for each time point, and presented after log_2_ transformation. All values are expressed as mean ± SE (*n* = 3). For each gene at each time point, Student’s *t*-test was used to compare values with controls, and asterisks denote changes meeting the predefined statistical thresholds of *p* ≤ 0.05 (*) and *p* ≤ 0.1 (**). Means for each gene were compared by the least significant difference (LSD) test (*p* ≤ 0.05), to show differences in gene expression dynamics, and statistically different means are denoted with different letters.

The expression profile of the analyzed *CYP72-2* gene showed just a slight induction compared to controls after 2 and 6 h of exposure (Figure 2), with the maximum value of log_2_FC = 2.2 after 2 h. A single exposure to FM-EO for 2, 4, 6, or 8 h resulted in a significant upregulation of all tested genes from subfamily 81D-like, with values of log_2_FC varying between about 4 and 7 for *81D11-1* and *81D1-1* genes compared to values in corresponding controls. The third gene, *81D1-2*, had consistently lower upregulation, however significantly different from controls after treatments of 2, 4 and 6 h. Notably, all three genes exhibited a similar expression pattern during the time-course after the exposure to FM-EO.

#### 3.1.4. Immunodetection of CYP81 Proteins in Potato Exposed to FM-EO

**Figure 3:**
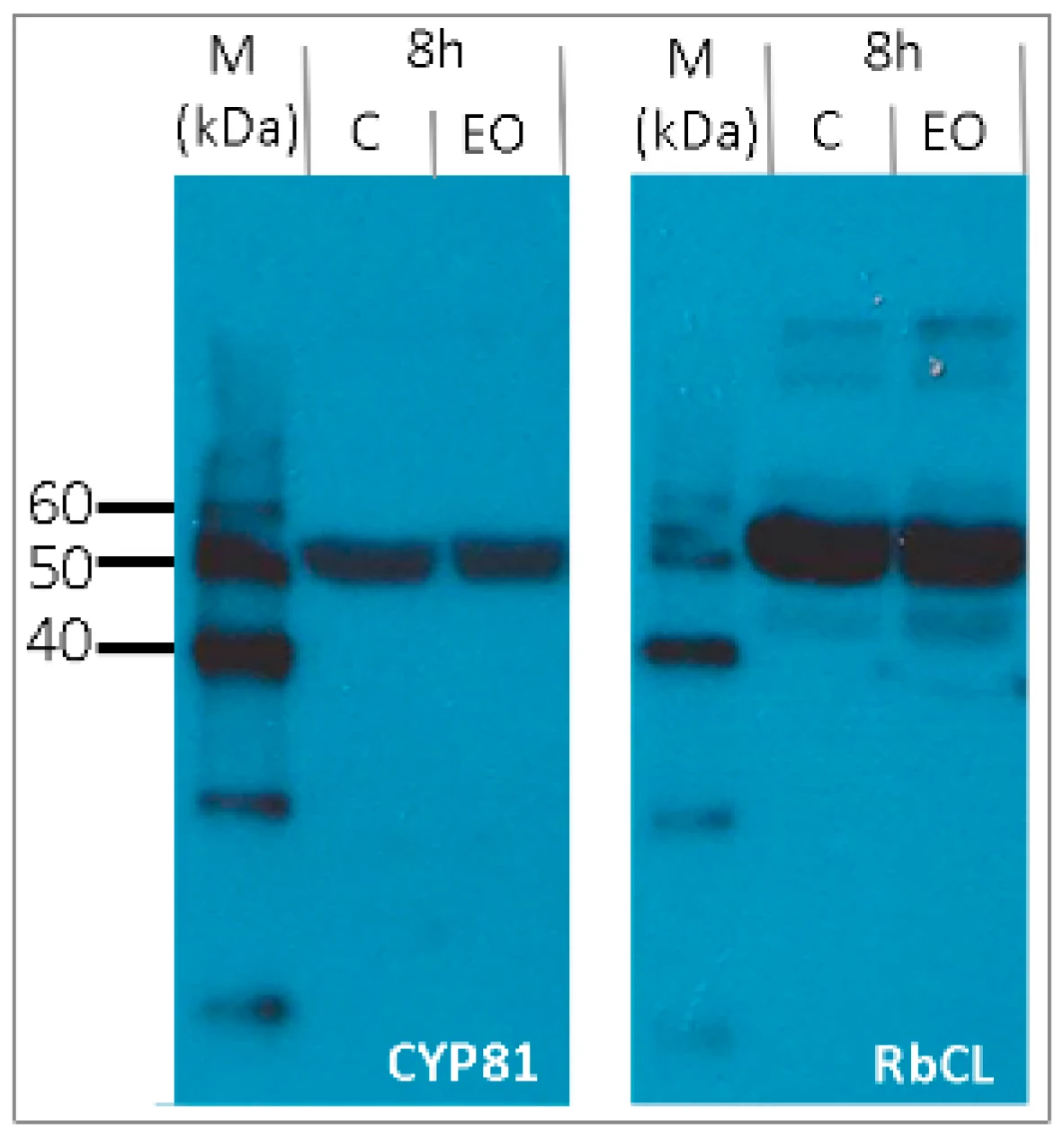
Western blot detection of CYP81 family enzymes in total soluble proteins of potato. The Rubisco large subunit (RbCL) was used as the loading control. M—MagicMark™ XP Western Protein Standard with protein bands of different size in kDa; C—control, EO—essential oil exposure for 8 h.

Western blot analysis revealed the protein band corresponding to the expected molecular weight of cytochrome P450 81 (CYP81) family enzymes (55.5 kDa) and the Rubisco large subunit (52–55 kDa) (Figure 3). The presence of CYP81 proteins was confirmed in both potato plants after 8 h of exposure and in control samples.

### 3.2. In Silico Characterization of Potato CYP81

Among the top eight highly DE CYP450-coding genes in EO-exposed potato were three representative transcripts of subfamilies 81D1-like and 81D11-like, the only members of these subfamilies in the whole cDNA microarray dataset exhibiting some unique characteristics (Table 5) and an inducible pattern of expression (Figure 2). Therefore, further in silico analyses were focused on the CYP81 subfamily.

#### 3.2.1. Extraction of CYP81 Sequences from Potato Genome

**Figure 4:**
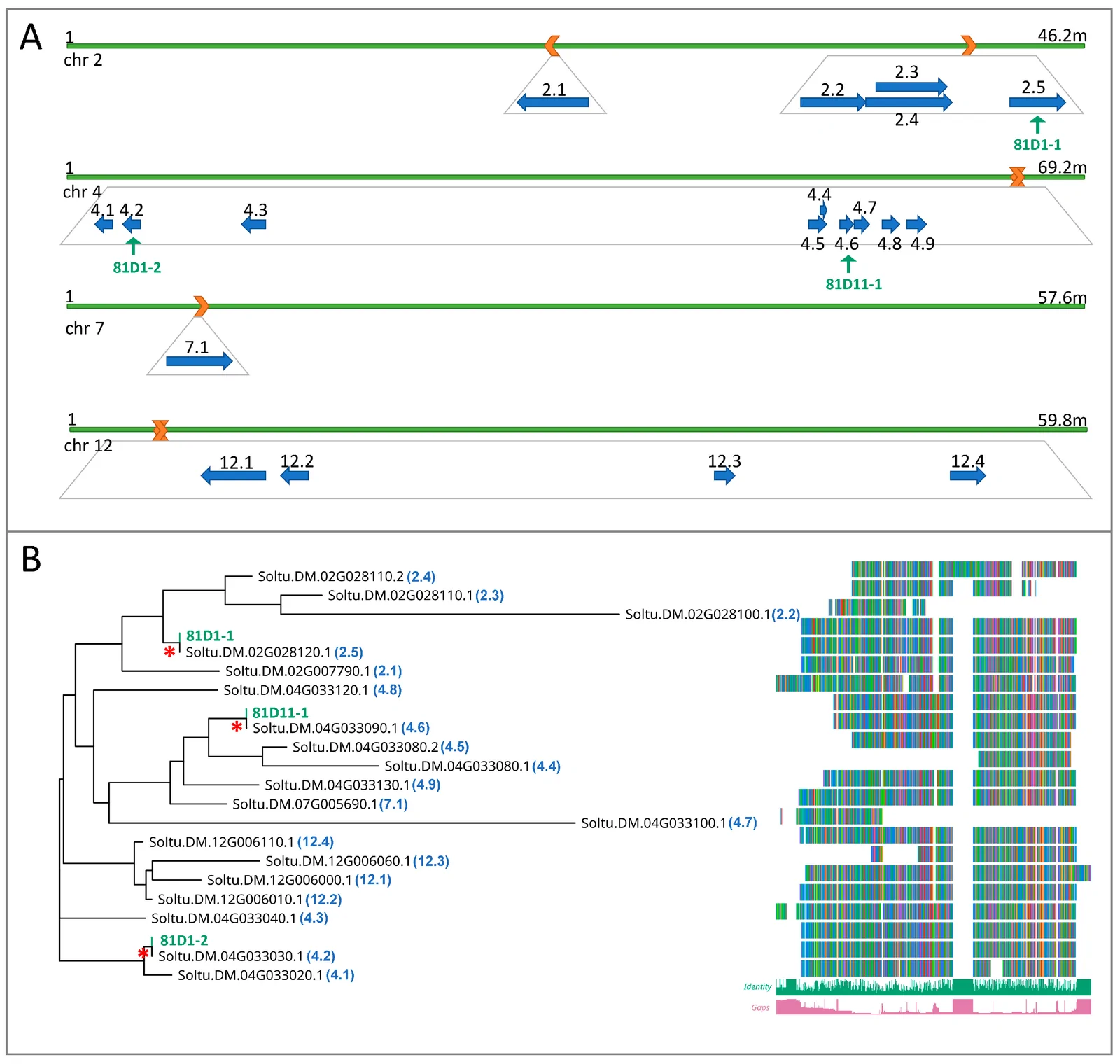
Identified *CYP81* genes in potato genome by the Spud DB Potato Genomics Resource (v6.1). (**A**) Chromosomal distribution and strain orientation of *CYP81* genes on chromosomes (chr) 2, 4, 7, and 12. Green lines represent the full length of a chromosome, orange arrows represent regions on the chromosome where *CYP81* genes are located and their orientation, and blue arrows represent *CYP81* sequence strand orientation and length relative to other genes in the same cluster. (**B**) Protein alignment and similarity of CYP81 members and DE *CYP81* genes (*81D1-1*, *81D1-2*, and *81D11-1*) analyzed in the study (red asterisks).

*CYP450* genes in the whole potato genome were identified using the Spud DB Potato Genomics Resource (v6.1), producing 694 sequences, of which 19 belonged to family 81 (Table S3). Eleven members belonged to the D subfamily, six to the K subfamily, and two to the F subfamily. These 19 sequences were identified on chromosomes 2, 4, 7, and 12 (Figure 4A).

To evaluate sequence similarity and cDNA microarray probe binding, members of the CYP81 family from Spud DB were aligned to the three *CYP81* sequences identified in the most DE microarray dataset (Table 1), labeled as *81D1-1, 81D1-2,* and *81D11*-1 (Figure 4B). Protein alignment showed 100% sequence identity between Soltu.DM.02G028120.1 and *81D1-1*, Soltu.DM.04G033030.1 and *81D1-2*, and Soltu.DM.04G033090.1 and *81D11-1*, suggesting those were the same predicted protein sequences in both genome versions.

According to InterPro classification (Table S4), 17 out of these 19 identified sequences belong to the Cytochrome P450 E-class I and to the Plant cytochrome P450 monooxygenase group. Protein lengths varied from 174 to 539 amino acids, while their molecular weights ranged from 20.30 to 61.63 kDa (Table S5). The theoretical isoelectric points (pI) were between 5.04 and 9.44, while grand averages of hydropathicity (GRAVY) values varied between −0.406 and 0.15. The GRAVY values were negative for 16 out of 19 protein members, indicating that they were proteins with predominantly hydrophilic regions, while a score close to 0 in all sequences suggested that there is a balance of amino acids comprising water-soluble and transmembrane regions, which mostly correlated with predictions.

#### 3.2.2. Predicted Function, Localization, and Structure of Potato CYP81 Proteins

**Figure 5:**
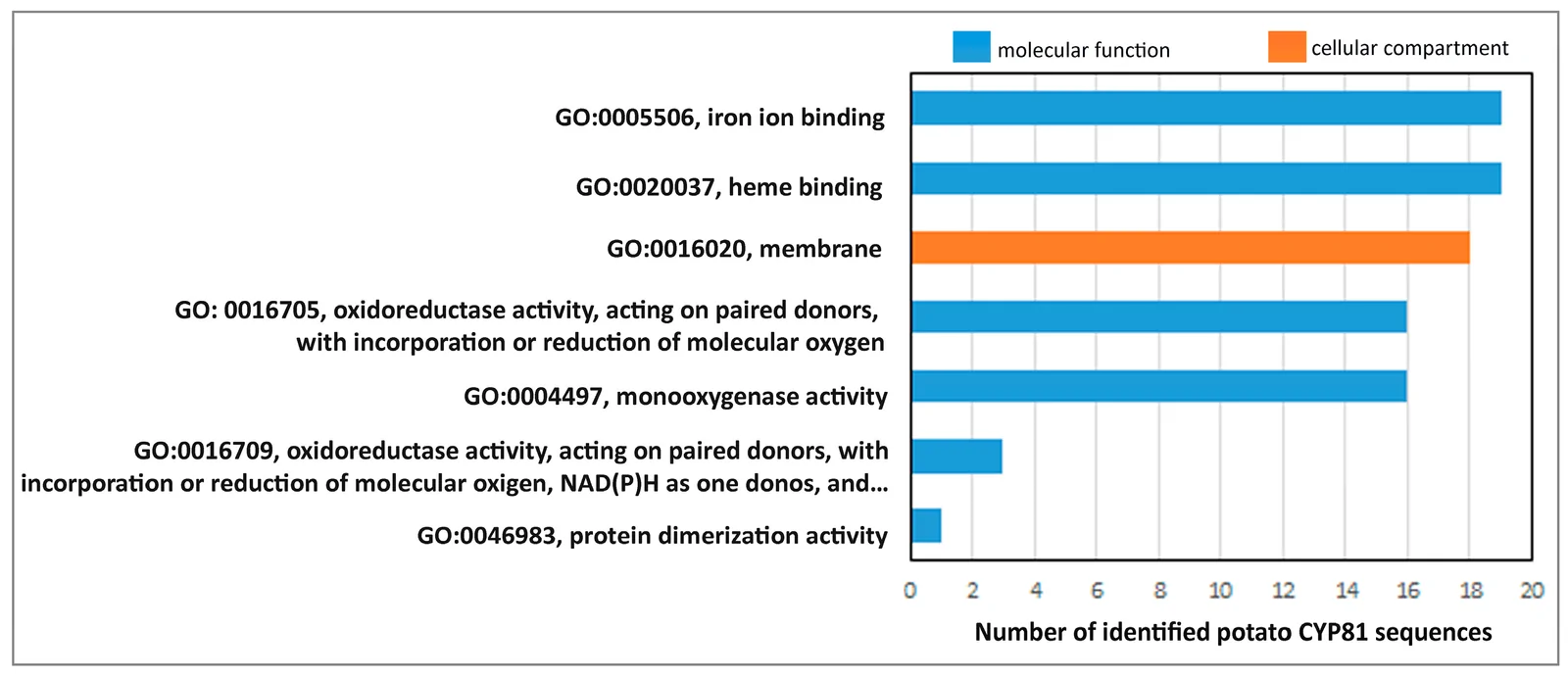
GO terms assigned to potato CYP81 family members by Blast2GO.

According to InterPro annotation, all sequences were in silico characterized by four molecular function (MF) GO terms: GO:0005506 (iron ion binding), GO:0020037 (heme binding), GO:0004497 (monooxygenase activity), and GO:0016705 (oxidoreductase activity, acting on paired donors, with incorporation or reduction of molecular oxygen). Additional Blast2GO annotation of CYP81 protein sequences also revealed one GO term related to cellular compartment (CC): GO:0016020 (membrane) and two more related to MF: GO:0016709 (oxidoreductase activity, acting on paired donors, with incorporation or reduction of molecular oxygen, NAD(P)H as one donor, and the incorporation of one atom of oxygen) and GO:0046983 (protein dimerization activity) (Figure 5). These terms confirmed the classical CYP450 functions and suggested a strong link between CYP81s and oxydoreductase and monooxygenase activity as well as membrane-binding position.

**Figure 6:**
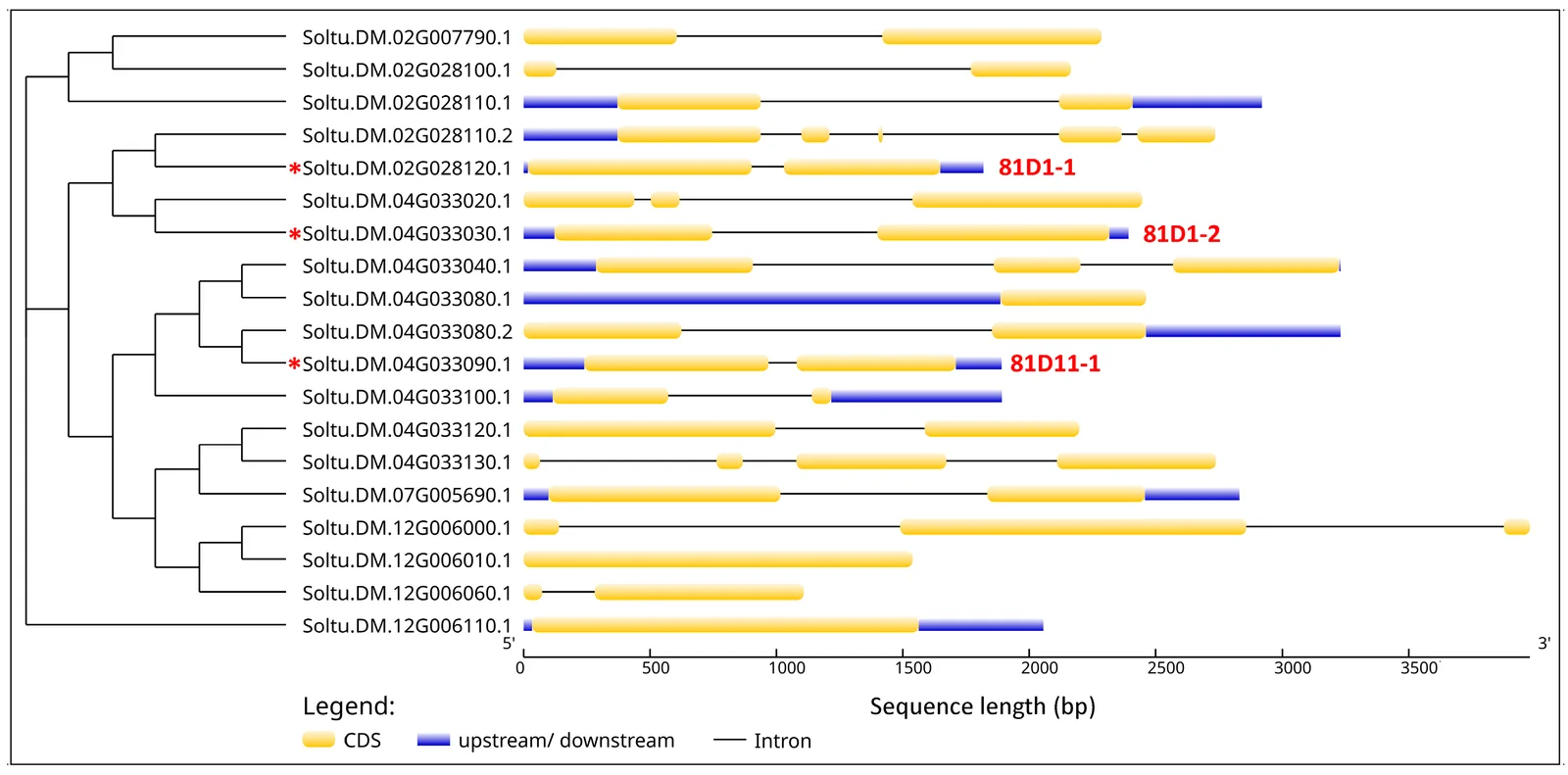
In silico predicted exon-intron structure of potato *CYP81* genes. Yellow rectangles represent exons, black lines indicate introns, and dark blue rectangles correspond to 5′ and 3′ UTR regions. The prediction was performed using the GSDS software. Red asterisks indicate the most expressed CYP81 sequences according to cDNA microarray and RT-qPCR (*81D1-1*, *81D1-2*, and *81D11-1*).

The predicted exon-intron structure of these *CYP81* genes revealed that the number of exons varied between one and five (Figure 6). Several genes (such as Soltu.DM.04G033080.2 and Soltu.DM.02G028110.1) contained extended 3′ UTRs, while others had shorter or absent 3′ UTR regions. Moreover, three sequences lacked introns (Soltu.DM.04G033080.1, Soltu.DM.12G006010.1, and Soltu.DM.12G006110.1).

#### 3.2.3. CYP81 Promoter Characterization

Analysis of computationally predicted promoter regions using the PlantCARE database revealed a diverse set of CREs (Table S6). Among the most abundant motifs, the core promoter elements TATA-box (503 occurrences) and CAAT-box (379) were found, both of which are commonly involved in basal transcriptional regulation. In addition, several regulatory motifs associated with TF binding were detected, including MYC (35 occurrences), MYB (34), and MYB-like sequences, with 12 occurrences. Multiple elements related to environmental and hormonal responses were also identified, such as the G-box (36 occurrences) and GT1-motif (16), both associated with light responsiveness, as well as ABRE (29), involved in abscisic acid signaling. Stress-related elements such as STRE (23 occurrences), ARE (21), and the W-box (10), which represents a WRKY TF-binding site, were also present. Hormone-responsive motifs, including the TCA-element (salicylic acid), TGACG-motif and CGTCA-motif denoting methyl jasmonate (MeJA) responsiveness, as well as ERE (ethylene response), were detected in lower frequencies. Several motifs with no annotated regulatory function in the PlantCARE database were also identified.

To further examine the regulatory potential of the analyzed promoters, the identified cis-elements were grouped according to their predicted biological functions. Light-responsive elements were the most abundant, with a total of 112 occurrences, followed by elements associated with ABA responsiveness (29), anaerobic induction (21), and MeJA responsiveness (18). Additional motifs related to TF binding (MYB binding sites), hormone signaling (gibberellin, salicylic acid, and auxin), stress responses (low temperature, mixed stress), and developmental processes (meristem, endosperm, seed, and cell cycle) were also detected with lower abundance.

**Figure 7:**
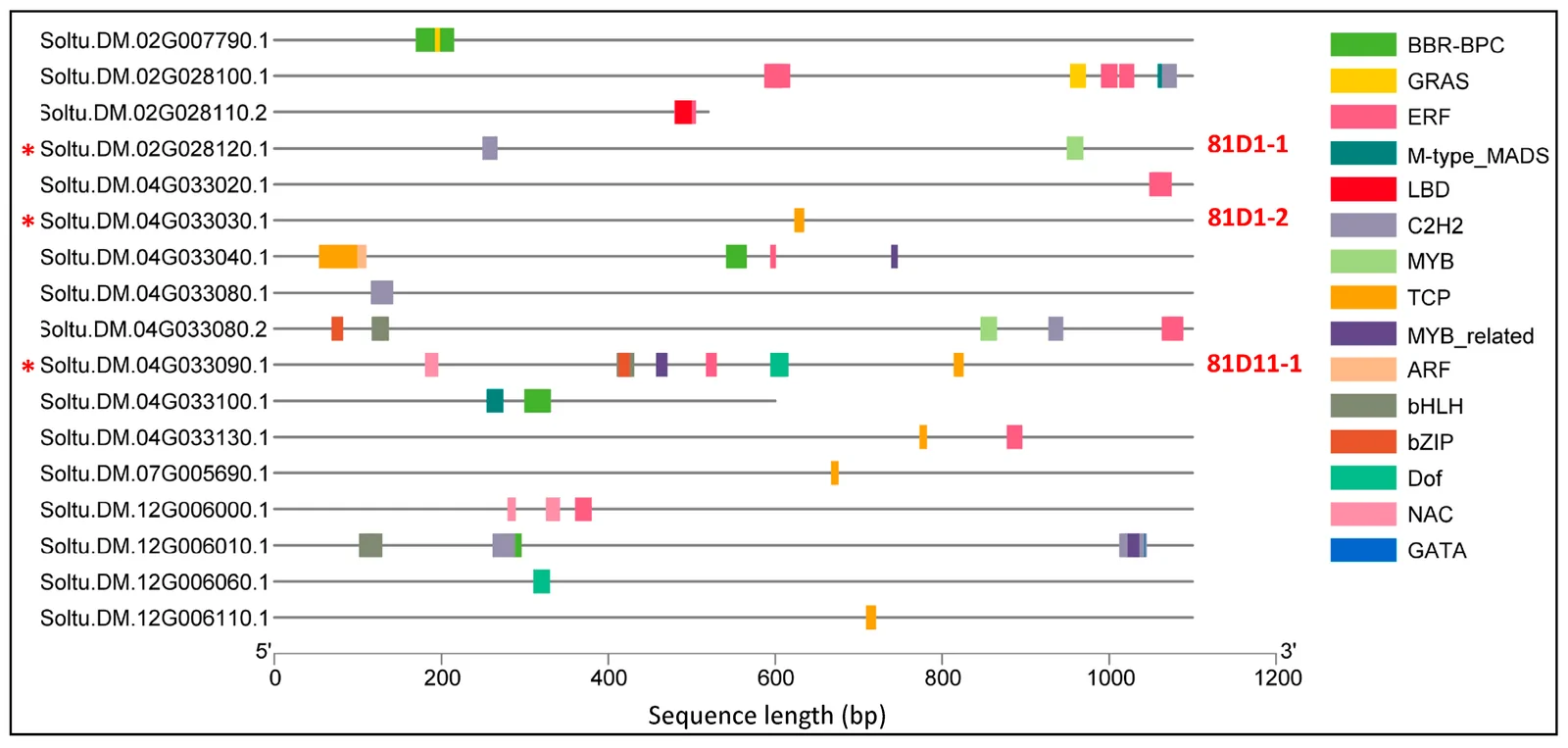
Predicted transcription factor binding sites in promoter regions of potato *CYP81* genes. Binding sites were identified using PlantRegMap and visualized with TBtools. Colored boxes represent predicted binding sites of different TF families along the promoter regions in the 5′–3′ direction. Red asterisks indicate the most expressed *CYP81* sequences (*81D1-1*, *81D1-2*, and *81D11-1*) according to cDNA microarray and RT-qPCR.

In total, 137 TF-binding sites belonging to multiple TF families were predicted for 17 sequences within analyzed promoter regions. The most abundant families were TCP (28 sites) and ERF (26), followed by BBR-BPC (17), C2H2 (14), bHLH (12), and bZIP (11) TF, while other families such as NAC, Dof, MYB, GATA, and GRAS were represented with lower frequencies. Multiple TF families capable of binding to the analyzed potato CYP81 promoter regions were distributed along the promoter regions of the analyzed genes (Figure 7).

#### 3.2.4. In Silico Predicted Structural Features of Potato CYP81 Proteins

The predicted structural features of potato CYP81 proteins indicated a predominantly α-helical fold typical of cytochrome P450 enzymes. Protein length varied considerably, ranging from 174 to 539 amino acids (Table S5), although the majority of members were approximately 450–540 amino acids long. In general, shorter protein sequences contained fewer secondary structure elements.

**Figure 8:**
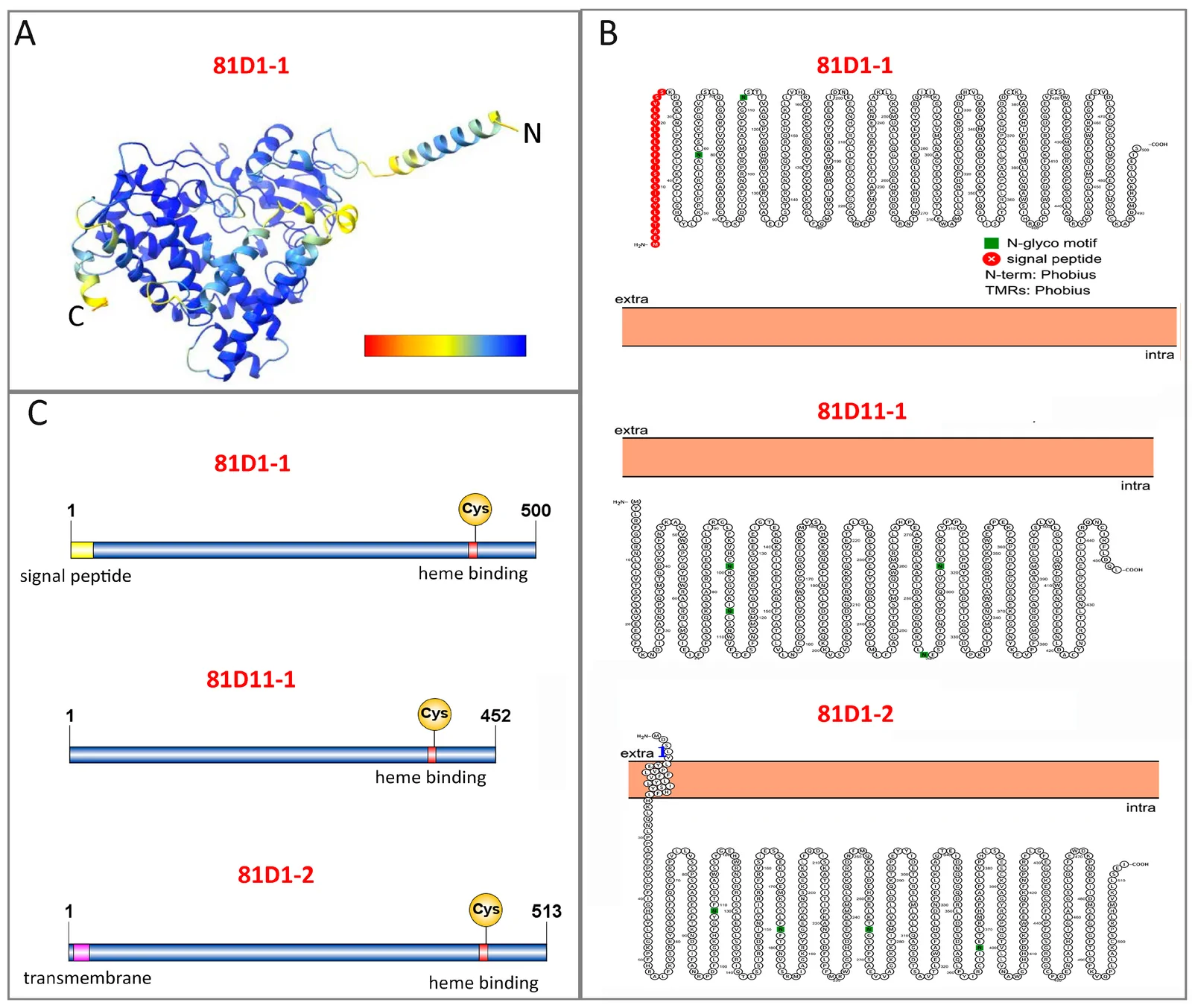
In silico prediction of three-dimensional (3D) tertiary structure (**A**), position relative to the membrane (**B**), and major protein domains (**C**) in selected potato CYP81 proteins (81D1-1, 81D1-2, and 81D11-1). The structural model was generated using AlphaFold and visualized in UCSF ChimeraX. The model was colored based on prediction confidence (pLDDT scores), ranging from blue (high confidence) to red (low confidence). The positions of the heme-binding domain (with conserved cysteine (Cys) residue indicated in dark yellow circle), signal peptide (light yellow), and transmembrane domain (purple) are shown along the selected CYP81 protein sequences.

The computationally predicted 3D structures of CYP81 family members showed the central region forming a compact globular core composed mainly of densely packed α-helices with a smaller contribution of β-strand segments. Most core regions exhibited high confidence, whereas peripheral loops and terminal segments showed lower confidence values. Representative 81D1-1 is shown in Figure 8. It consisted of a well-defined high-confidence core with an elongated terminal helix extending from the compact central domain and displaying slightly reduced prediction confidence. CYP81 sequences annotated by InterPro were characterized by several representative domains: a cytochrome P450 cysteine heme–iron ligand signature (ProSitePatterns PS00086), signal peptide, and transmembrane domains (Table S4). A heme–iron ligand signature was detected in 16 sequences, signal peptide was present in 2 sequences, and the transmembrane region was predicted in 8 and 6 sequences, depending on the prediction method. The presence of transmembrane and extracellular regions in these transcripts makes them especially interesting in the context of VOC interaction research. Representative members of CYP81 predicted to be extracellular, intracellular, or with transmembrane domains are represented in Figure 8. Potato CYP81 proteins confirmed to have a conserved heme-binding domain were characterized by the conserved cysteine (Cys) residue within the FxxGxxxCxG motif (Figure 8). The heme-binding domain was predominantly located in the C-terminal region, most frequently between positions ~380 and 480 aa, while shorter sequences either contained it proportionally earlier in the sequence or lacked the motif. Phobius analysis indicated that eight of the potato CYP81 proteins contain predicted transmembrane domains, predominately located on chromosome 4. Five of CYP81 proteins possess a single N-terminal transmembrane helix positioned within the first ~50 amino acids, suggesting membrane anchoring typical for plant CYP450 enzymes (Figure 8).

#### 3.2.5. In Silico Predicted Potato CYP81 Protein Interactions

Using the model plant Arabidopsis as a background, 19 potato CYP81 proteins were mapped to 7 related Arabidopsis proteins (Figure S1, Table S7). The closest 10 potential interacting proteins revealed a functionally heterogeneous but biologically coherent cluster (Figure S1, Table S8). The predicted associations were enriched in other interacting cytochrome P450 enzymes involved in fatty acid modification, cuticle biosynthesis, and oxylipin metabolism; proteins linked to auxin signaling pathways; enzymes associated with lipid turnover and peroxisome-dependent metabolic pathways; and proteins related to stress responses. Interacting CYP450 proteins included four proteins from family 86 (CYP86C1-4) and one protein from family 94 (CYP94B2), while other connections comprised of auxin-responsive endogenous peptide 1 (AREP1), GDSL esterase/lipase (CDEF1), purple acid phosphatase 18 (PAP18), peroxisome biogenesis protein 19-1 (PEX19-1), and one uncharacterized protein (F8L15_160). Together, these findings point toward a role of CYP81 proteins in coordinating lipid-derived specialized metabolism with hormone-regulated stress and developmental processes. It should be noted, however, that these STRING associations reflect functional links derived from gene co-occurrence, protein homology, and text mining from curated databases, which offer indirect evidence; therefore, experimental validation would be needed to confirm these interactions. The additional GO enrichment analysis of proteins in the interaction network demonstrated well-known and expected GO terms related to CYP81 family (heme binding, iron ion binding, monooxygenase and oxydoreductase activity) but also revealed new GO terms describing heterocyclic compound binding, organic cyclic compound binding, metal ion binding, and catalytic activity (Figure S2).

### 3.3. Enzyme-Substrate Computational Prediction to Estimate the Biological Significance of Potential Interactions Between CYP81s and Selected Essential Oil Constituents

To estimate potential enzyme–substrate interaction, the protein sequences of the CYP81 family were modeled against eight significant constituents of FM-EO. These included terpinolene, limonene, (*E*)-caryophyllene, and (*Z*)-β-ocimene as the major components, as well as piperitone, (*Z*)-tagetone, (*E*)-tagetone, and dihydrotagetone as the most promising putative targets.

**Figure 9:**
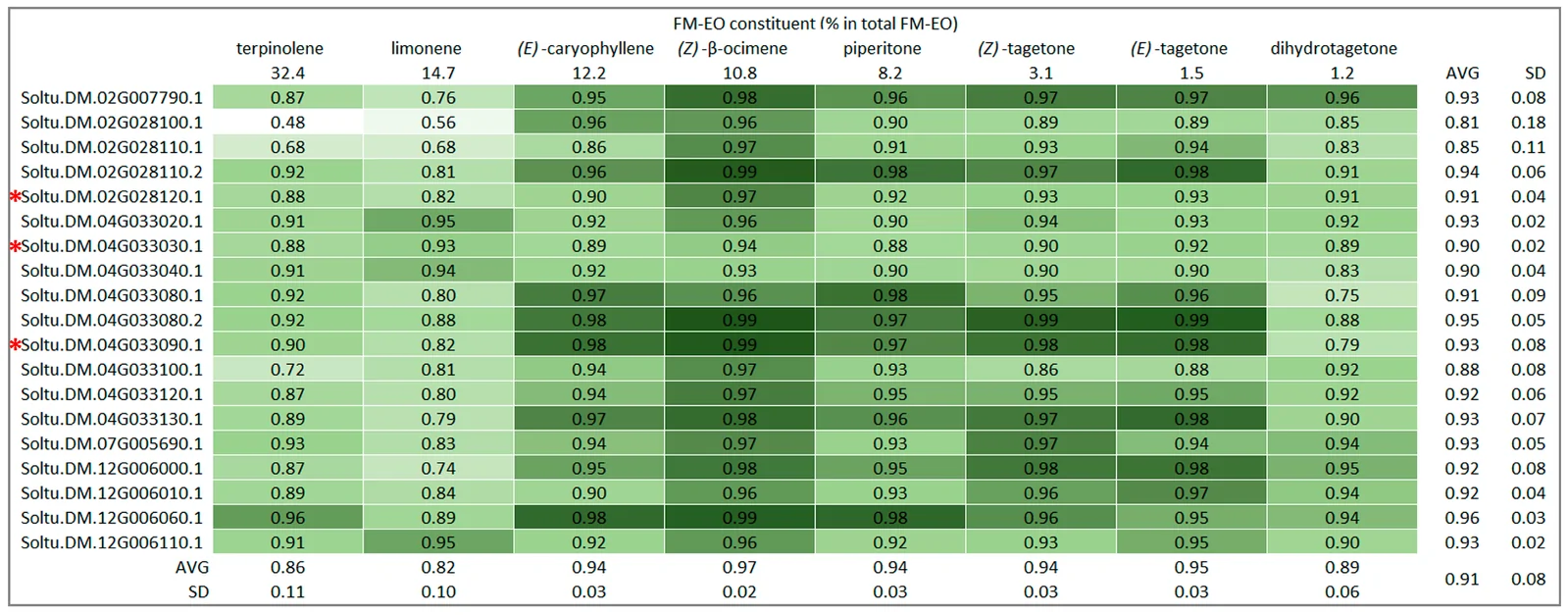
Enzyme–substrate interaction of potato CYP81 proteins with FM-EO components, predicted by P450Rdb. Red asterisks indicate the most expressed CYP81 sequences according to cDNA microarray and RT-qPCR (*81D1-1*, *81D1-2*, and *81D11-1,* respectively). Higher interaction scores are represented by darker green shades.

The prediction results, obtained using the P450Rdb machine learning model, indicate the likelihood of interactions between a CYP450 enzyme and the substrate molecule based on previously identified biological patterns in CYP450 enzymes (Figure 9). Looking at the combination of all the compounds and protein sequences, there was a favorable average interaction score of 0.91 ± 0.08 (AVG ± SD), while the averages across one compound or one protein sequence varied significantly. (*Z*)-β-ocimene showed the best interaction score (0.97 ± 0.02) with all of the potato CYP81s, while the protein Soltu.DM.12G006060.1 showed the best score across all tested compounds (0.96 ± 0.03).

To estimate spatial orientation and fit of the FM-EO compounds to proteins of the potato CYP81 family, blind docking analysis was performed, with heme domain added from similar plant CYP81 sequences with deconvoluted structures. Out of 19 CYP81 sequences, 16 had a heme-binding domain (FxxGxxxCxG motif). Those sequences were aligned to known X-ray crystal plant protein structures, and heme was transferred from the most similar CYP450 structure to our CYP81 model accordingly. Ten out of 16 CYP81 proteins that contained heme-binding regions had the heme group in the favorable position toward the cysteine residue in the heme-binding region. In those sequences, the distance between Fe ions in heme and SG (the gamma-sulfur) atoms in the cysteine residue was predicted to be less than 3.2 Å, which was an indication of correct assembly of the heme-binding pocket.

All CYP81 protein 3D structures with aligned heme group were used for cavity-detection guided blind docking, along with ligand sequences of five chosen essential oil compounds: (*Z*)-β-ocimene, piperitone, (*Z*)-tagetone, (*E*)-tagetone, and dihydrotagetone (Table S9).

Preliminary docking analysis suggested a potential binding interaction within the large pocket near the heme prosthetic group. The binding prediction included only the interaction of amino acids and ligands, but the position of heme was used to assist the prediction of the pocket size and to choose the ligand position closest to heme. The predicted Vina scores varied from −6.9 kcal/mol (for piperitone and Soltu.DM.04G033030.1) and −4.3 kcal/mol (Table S9). Among the tested compounds, piperitone had the highest average score across all CYP81 sequences (−6.16 ± 0.53 kcal/mol), while the sequence Soltu.DM.02G007790.1 showed the highest average score across all tested compounds (−6.58 ± 0.13 kcal/mol).

**Figure 10:**
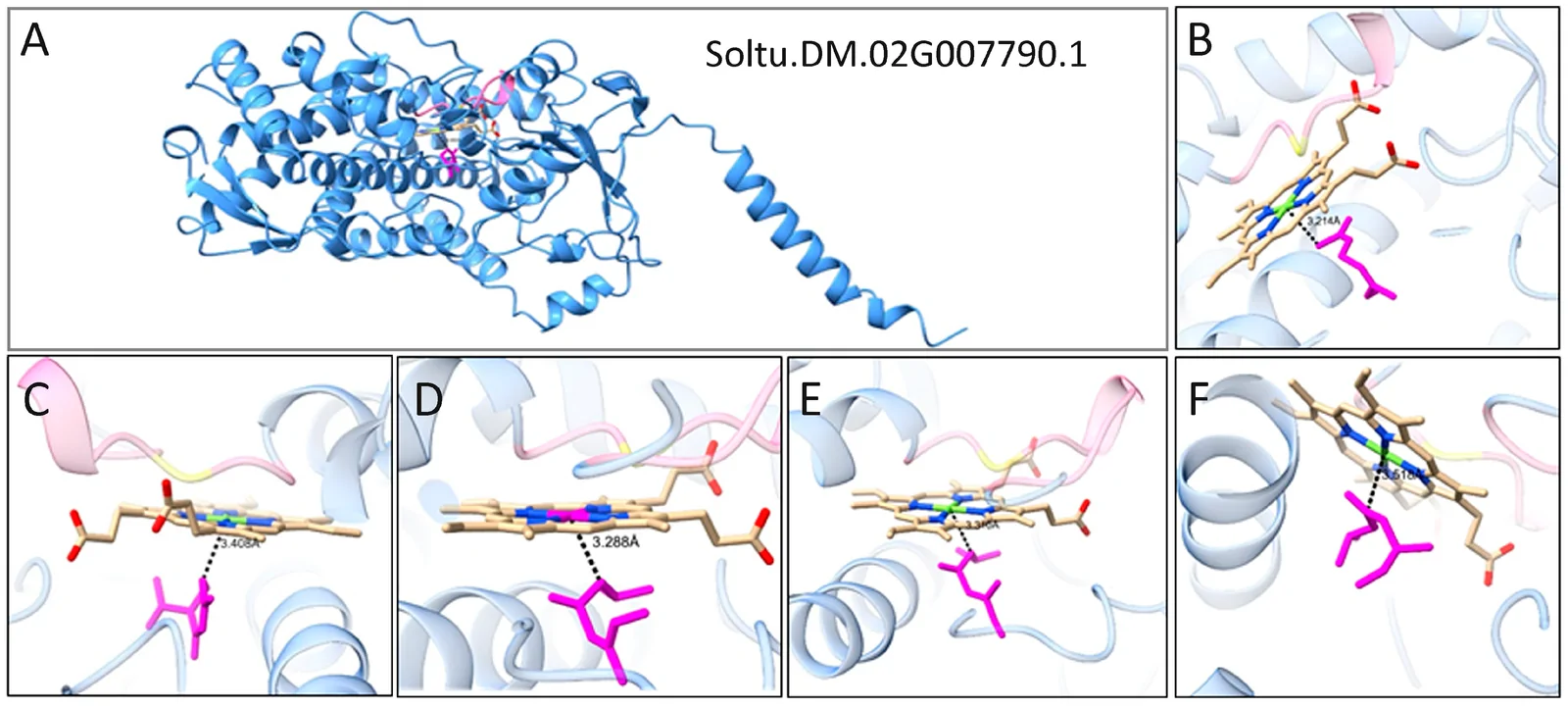
Predicted docking of potato CYP81s with French marigold essential oil compounds. (**A**) 3D structure of Soltu.DM.02G007790.1 with added heme and ligand (piperitone, in purple). Best docking position for each of the compounds with measured minimum distance between Fe ion and ligand and presented for (*Z*)-β-ocimene (**B**), piperitone (**C**), (*Z*)-tagetone (**D**), (*E*)-tagetone (**E**), and dihydrotagetone (**F**).

The 3D structure of Soltu.DM.02G007790.1 with added heme is represented in Figure 10A, demonstrating a fairly globular structure, with the heme region towards the inside of the structure, and with the cavity volume of 2076 Å3. The distance measured between the Fe ion and ligand in the best docking positions for each of the compounds, illustrated on Figure 10B–F, was, respectively, 3.214 Å for (*Z*)-β-ocimene, 3.408 Å for piperitone, 3.288 Å for (*Z*)-tagetone, 3.310 Å for (*E*)-tagetone, and 3.518 Å for dihydrotagetone, suggesting a possible catalytic position.

The preliminary docking analysis thus suggested piperitone as the compound most likely to interact with potato CYP81, and the protein encoded by Soltu.DM.02G007790.1 as the CYP81 protein most likely to be able to interact with ligands through a heme group and neighboring amino acids in the sizable binding pocket.

## 4. Discussion

### 4.1. Potato CYP81 Expression During FM-EO Treatment

In the whole-transcriptome microarray data of potato exposed to FM-EO, around 10% of microarray probes were found to be differentially expressed (DE), suggesting that a massive transcriptional reprogramming occurred. Moreover, around a third of detected *CYP450* transcripts were significantly DE, highlighting the substantial impact of this treatment on multiple family members. The majority of DE potato CYP450s belonged to the CYP71-like clan, which corroborated previous findings that CYP71 is the most prevalent clan in the potato genome [26,28]. The large majority of potato DE CYP450 members were further classified as E-class group I, while CYP72A219-like was the dominant subfamily. The highest upregulated transcripts belonged to subfamilies 81D1-like, 81D11-like, and CYP72A219-like (fc from 9.38 to 31.95) suggesting that these subfamilies were the most responsive to FM-EO treatment and possibly involved in volatile signaling and/or detoxification.

The four representatives of subfamilies CYP72A219-like, 81D1-like, and 81D11-like (*CYP72-2, 81D1-1*, *81D1-2*, and *81D11-1)* were chosen to study the dynamic of gene expression following exposure to FM-EO volatiles. The CYP72A219-like subfamily member, *CYP72-2*, gene expression demonstrated little effect of FM-EO. In contrast, members of 81D1-like and 81D11-like revealed strong induction with FM-EO treatments. In addition, immunodetection of CYP81 proteins in potato demonstrated the presence of these proteins. RT-qPCR confirmed the transcriptional induction of the selected CYP81D-like genes, whereas immunoblotting confirmed the presence of CYP81-immunoreactive proteins.

The CYP81 family is a distinct family within clan 71, well known for its role in herbicide metabolism and resistance [9,11,22]. Many members, especially the CYP81D and CYP81E subfamilies, are strongly induced by abiotic stresses, such as high light intensity and drought [51], high salinity [52], or xenobiotic treatment [53,54,55]. In potato, a recent RNA-seq study found two CYP81D genes: *StCYP76* (corresponding to *81D1-1* from our study) and *StCYP237*, and one CYP81K gene *StCYP239* (corresponding to *81D11-1* from our study) to be upregulated by salt stress, with *StCYP76* displaying especially prominent induction at 12 h [26]. On the other hand, some members of the CYP81 family in monocot species showed a constitutive overexpression in populations resistant to herbicide treatment, which was not further elevated by the herbicide treatment itself [56,57]. Other subfamily members were shown to be induced by wounding [55,58,59] or fungal pathogen infection [53].

*CYP81D11* gene expression in Arabidopsis has especially been shown to be induced by different reactive chemicals, like TIBA and BOA, including also xenobiotics such as TNT [53]. It was also highly inducible by some volatile compounds, like methyl jasmonate (MeJA) and its derivatives [53], although the activation pathways of xenobiotics and MeJA are likely independent at the *CYP81D11* promoter level [54]. For example, cis-jasmone (CJ), a plant-derived floral volatile, released in response to herbivore attacks to attract predators of herbivores, was reported to be a strong inducer of *CYP81D11*. When added externally, wild-type plants treated with CJ exhibited this attraction, while knockout mutants of *CYP81D11* failed to do so, demonstrating a role of this gene in indirect induced defense mechanisms [53]. Additionally, exposure to β-cyclocitral induced large-scale transcriptomic changes, inducing a range of genes related to defense and detoxification (such as *GST* and glucosyl transferase *UGT* genes), but the most strongly induced gene under all exposure conditions was *CYP81D11*, showing log_2_fc > 8, suggesting that this gene was a key component in plant chemical stress tolerance, especially in high-light environments [51].

Moreover, CYP450s with demonstrated inducibility by foreign compounds (such as CYP76B1 in Jerusalem artichoke) exhibited both broad substrate ranges and high catalytic efficiency, indicating evolutionary adaptation specifically for diverse xenobiotic response [60]. While transcriptional induction alone does not confirm catalytic involvement in FM-EO metabolism, the marked responsiveness of *CYP81D* genes and the predicted structural compatibility of CYP81 proteins with FM-EO constituents suggest that this subfamily is a promising candidate for future functional characterization.

### 4.2. Genomic Architecture and Classification of Potato CYP81

Although *CYP72-2* transcripts were also differentially expressed in the microarray analysis, subsequent RT-qPCR showed only modest responsiveness to FM-EO. Therefore, the discussion focuses primarily on CYP81 family members, whose induction was considerably stronger. The number of *CYP450* genes belonging to the *CYP81* family varies between different plant species due to lineage-specific duplication events, often referred to as a “CYP bloom” [61]. According to the 2020 version of the potato genome, 19 *CYP450* genes and predicted proteins were characterized as family 81 CYP450s, belonging to subfamilies CYP81D (11 members), CYP81K (6 members), and CYP81F (2 members). Similarly, in Arabidopsis, 18 *CYP81* loci were identified in subfamilies 81D (10), 81F (4), K (2), G, and H [62], coding for 16 CYP81 proteins [63]. This expansion of gene copies within a CYP450 family or subfamily is frequently associated with adaptive responses, new metabolic pathways, and speciation [61]. In one such example, a multiple-herbicide-resistant weed *Echinochloa phyllopogon* possessed at least 12 *CYP81A* genes conferring herbicide resistance, including three considered to be frameshift-type pseudogenes [56]. The variations in gene copy number and gene orientation suggested that *CYP81* clusters were dynamic and species-specific, and tended to occur by tandem duplication [52,61]. Potato *CYP81* genes were found in several clusters on chromosomes 2, 4, and 12, while one gene copy was also present on chromosome 7. Similarity comparison suggested that sequences on chromosome 12 represented a more homogenous cluster all belonging to the *CYP81D* subfamily, while the most diversified group of potato *CYP81s* was present on chromosome 4, and the *CYP81* sequence on chromosome 7 seemed to be a duplication event with some divergence. Localized tandem gene duplication and significant clustering across chromosomes is a prominent characteristic of all potato *CYP450* genes [26].

The gene structure of potato *CYP81* genes is defined by characteristics common to A-type CYP450s, as well as specific evolutionary patterns unique to this family. A-type CYP450s commonly have fewer exons and introns than non-A-type CYP450s, and most of them have a single highly conserved intron [10]. In potato, 11 out of 19 *CYP81* sequences contained only one intron. All three highly expressed *CYP81* genes in potato exposed to FM-EO have one intron splitting two CDS regions, with present 5′ and 3′ UTR regions. An interesting evolutionary feature of some members of the *CYP81* family is “intron-less bloom”, occurring during gene duplication, and widespread within several Arabidopsis CYP450 families [63,64]. Indeed, some potato *CYP81* family genes completely lack introns, suggesting that the duplication of these genes occurred by these retro-position events [64]. Like in Arabidopsis [65], potato CYP81 family members were characterized by up to five exons.

### 4.3. Roles of CYP81s in the Network of Detoxification and Stress Signaling

The CYP81 family of enzymes is a highly versatile CYP450 family, shown to be involved in the biosynthesis of specialized metabolites like terpenoids, isoflavones, and alkaloids; in the response to different stresses like drought, salinity, and heavy metals; but also in the detoxification of xenobiotics, especially herbicides [9,10,13]. The CYP81A subfamily was extensively studied in monocot grass crops and weeds for directly detoxifying herbicides [9,10,66]. While there is some evidence that other CYP81 subfamilies could be involved in some dicots, like subfamily CYP81E in soybean [67] and subfamily CYP81B in Jerusalem artichoke [10] and tobacco [64,68], dicots mostly rely on other CYP450 families, like CYP72, and on GST enzymes to detoxify against herbicides [25]. Herbicide sensitivity in crops can be additionally alleviated by herbicide safeners, synthetic chemicals that proactively protect grass crops from herbicide injury without reducing the herbicide’s effectiveness against target weed species [69,70]. Safeners induce the entire detoxification network, but primarily affect CYP450s by acting as potent transcriptional inducers [70], and the CYP81 family has been shown to be the most affected in Arabidopsis [10].

Functional GO term analysis of potato CYP81s revealed vital molecular function terms related to monooxygenase and oxidoreductase activity, along with heme and iron ion binding, confirming the capacity of potato CYP81 family for classical CYP450 functions. Additional terms related to the membrane compartment and protein dimerization activity suggested possible associations with other proteins in the network. The wider network of protein–protein interactions revealed the likely connection of CYP81 proteins with other CYP450 proteins, possibly coordinating lipid metabolism with stress and development responses. Functional GO terms in this wider network of protein interactions expanded the classical CYP450 functions to include heterocyclic and organic cyclic compound binding, possibly suggesting that the interaction of CYP81 proteins with VOCs is accomplished via proxy network of other interconnected proteins. Many CYP450 enzymes, including those from the CYP81 family, often form organized multienzyme clusters—metabolons—to facilitate efficient metabolite flux and prevent the leakage of toxic intermediates [71].

A central response in both abiotic and biotic stress is ROS accumulation and signaling. Xenobiotic exposure almost universally triggers an oxidative burst and ROS accumulation within plant cells by inhibiting the antioxidant defense system, producing reactive and harmful intermediates and causing mitochondrial dysfunction [5]. In potato plants exposed to French marigold EO, the significant accumulation of short-lived •OH radicals indirectly proved the uptake of EO volatile compounds by the cells of the receiving plants [24]. Owing to its high sensitivity for detecting transient free radicals, even at low concentrations and despite their extremely short lifetime (~10 ns), electron paramagnetic resonance (EPR) spectroscopy revealed a rapid increase in •OH radical levels shortly after potato plants were exposed to FM volatiles. Consequently, as early as 2 h after exposure, a pronounced increase in the oxidized form of glutathione (GSSG) indicated the involvement of the glutathione redox cycle in the antioxidant scavenging of the newly generated free radicals. Similarly, in weed barnyard grass, EO treatment has been shown to upregulate antioxidant enzymes like catalase (CAT), peroxidase (POD), and superoxide dismutase (SOD) in a time-dependent manner [72]. In potato plants, the increase in ROS levels was accompanied by a decrease in glutathione abundance and a significant accumulation of GST transcripts [24]. Because GSTs generally conjugate substrates containing reactive functional groups, the observed induction of GSTs raises the possibility that at least a proportion of absorbed volatile compounds first undergo CYP450-mediated oxidation before glutathione conjugation.

The ROS signal, generated upon volatiles entry, activates stress-responsive transcription factors, which then simultaneously upregulate antioxidant enzymes (as it was shown for GSH in potato), but also other Phase I/II detoxification enzymes, CYP450s included [70,73,74]. It has been shown that xenobiotic herbicides cause rapid generation of ROS [5,70], which in turn acts as a signal leading to the upregulation of specific CYP450 families, like CYP81s [9,70], acting then directly or indirectly to detoxify herbicides [9,10,56]. Similarly, VOCs from French marigold induced fast sequential ROS accumulation in potato [24], while the same plants after 8 h of exposure exhibited significant expression of CYP450 representatives of the CYP72 and CYP81 families.

At least some members of the CYP81 family, like CYP81D11 in Arabidopsis, seem to act as components of a stress signaling pathway that boosts physiological efficiency by enhancing CO_2_ assimilation, thereby reducing the energetic imbalance that leads to the overproduction of damaging reactive oxygen species, rather than having a direct detoxification role [51]. Other members, like TaCYP81D5 in bread wheat, act as an important component of ROS signaling during abiotic stress, involving Zat12 transcription factor, which upregulates key antioxidant enzymes, improving a plant’s ability to scavenge excess ROS [9,52]. In addition, *CYP81E9*, a gene involved in isoflavone and flavonoid biosynthesis in Arabidopsis, may improve resistance by lowering and scavenging ROS [10].

Promoter-region analysis of the potato CYP81 family revealed elements related to environmental and hormonal responses, stress signaling, developmental responses, and reaction to light. These findings corroborated the broader CYP450 promoter analysis in potato [26,28], and supported the role of the CYP81 family in the regulation of growth, development, and stress in potato. In addition, several TF-binding motifs were detected in potato *CYP81* promoter regions, like bZIP, ERF, bHLH, MYB, and GRAS, involved in chemical signaling and detoxification pathways. TFs from bZIP and GRAS families are known to form complexes as functional partnership to induce the detoxification genes [54]. Several studies suggested a close relation between bZIP family of TFs, especially TGA TFs, with activation of a xenobiotic detoxification program [54]. In Arabidopsis, the induction of CYP81D11 by xenobiotics or apocarotenoids is strictly dependent on the TGAII–SCL14 complex, where TGAII (bZIP family) binds directly to *CYP81D11* promoter, and SCL14 (GRAS family) interacts with TGA, acting as a coactivator [51,54]. On the other hand, activation of the same gene by jasmonates, which are often induced during stress responses including xenobiotic treatment, involves a MYC2 TF, a core transcription factor in the jasmonate signaling pathway belonging to the bHLH family [54]. These two signals—jasmonates and xenobiotics—appear to use different combination of “switches” at the promoter level, where jasmonates require both MYC2-binding and TGA-binding elements to be present, unlike xenobiotics, which require only TGA-binding elements. However, if a MYC2-binding element is present alongside TGA, the chemical signal of xenobiotics is amplified, leading to a stronger response [54]. ERFs are known mediators of the ethylene signaling pathway, which has been shown to regulate detoxification enzymes (CYP450s and GSTs) during oxidative and chemical stress, potentially stabilizing cell membranes against lipophilic volatiles [74]. Some ERF-type TFs, in combination with CYP450s like WXP1 in *Medicago sativa*, can contribute to lowering chemical stress by enhancing the cuticular barrier and preventing the entry of xenobiotics [9]. MYB transcription factors respond to oxidative stress caused by xenobiotics, through upregulation of the antioxidant compounds synthesis [71], and are involved in ABA-JA stress signaling [53]. This motif, together with C2H2, has been found in Soltu.DM.02G028120.1, corresponding to the XM_006339781.1 sequence analyzed in this study, which was the most expressed *CYP81* gene in RT-qPCR, and was among the top ten DE microarray sequences. Interestingly, the other inducible sequence Soltu.DM.04G033090.1 (XM_015305409.1) has the highest number of predicted TF-binding sites (7), suggesting broad transcriptional plasticity, including responsiveness to volatiles.

Taken together, transcriptomic induction, promoter architecture, and previous evidence for ROS- and GST-mediated responses support the hypothesis that CYP81 enzymes may participate in an integrated detoxification network activated after FM-EO exposure.

### 4.4. In Silico Potential of Potato CYP81 Family for VOC Processing

Most plant CYP450s are membrane-bound enzymes anchored in the endoplasmic reticulum membrane, or less often in the chloroplast membrane, with a N-terminal hydrophobic membrane helix, while the remainder of the protein is in the cytosol [11,14]. Potato CYP81s indeed contain a balance of amino acids comprising water-soluble and transmembrane (TM) regions, and the majority of them have a predicted TM region or helix and associated GO compartment terms related to the membrane. Two *CYP81* sequences were predicted to be extracellular and contain signal peptide sequences, of which *81D1-1* is one. The prediction of TM and signal regions on the same sequence possibly can be an artifact within SignalP and transmembrane-helix predictions, which can falsely classify a hydrophobic transmembrane helix at the beginning of the sequence as a secretory signal peptide, especially in plants [75].

CYP450s bind their diverse ligands in a buried, hydrophobic active site, which is accessed through a substrate access channel, surrounded by α-helices (labeled A–K) and a β-pleated sheet [62]. In potato CYP81 proteins, a compact globular core was predicted, composed mainly of α-helices and a smaller number of β-strand segments, sharing a common 3D architecture of all CYP450 proteins [11]. While in general the 3D structures of CYP450s are quite conserved, the observed variations in the regions surrounding the catalytic site could be responsible for the perceived diversity of possible substrates, especially in some CYP450 groups, where variations in the length and position of these loops and helices can create cavities of different dimensions and architectures, allowing the enzymes to accommodate and position a diverse array of chemical structures over the fixed heme plane for catalysis [62]. A higher number of CYP450s in plants serve biosynthetic rather than detoxification roles [18,76]. In addition, one could speculate that biosynthetic CYP450s have evolved more stringent substrate selectivity, unlike xenobiotic-specialized CYP450s that possess active site architectures accommodating diverse chemical structures. While some plant CYP450 families show substrate-selectiveness, the CYP81 family is especially known to accept very diverse substrates, serving as an example of catalytic flexibility and substrate permissiveness [17]. This high level of substrate promiscuity is important in both natural defense mechanisms of plants and in their ability to resist synthetic chemicals, allowing them to metabolize a diverse range of endogenous and exogenous (xenobiotic) compounds. The CYP81 family is noted for its “blooming-prone” nature, undergoing significant expansion that allowed its members to participate in widely different metabolic pathways, including detoxification [61].

CYP450s can directly act on EO components, like terpenes, as shown during their biosynthesis in plants [64], but also on EO components during detoxification pathways in insects, herbivores, and humans [77,78]. It has been shown that EO treatment and plant allelochemicals activate detoxification pathway and transcriptomic changes, especially upregulation of distinct sets of *CYP450* genes, in several insect species [78,79,80,81,82]. EO components were demonstrated to be processed by plants as well [3], and this detoxification was proposed to be, at least in part, due to the action of CYP450 enzymes [83]. EO constituents have been shown previously to have herbicidal effects [3,84], while their derivatives from detoxification pathways in plants were shown to be less toxic than the parent compounds [83].

CYP450 enzymes metabolize lipophilic xenobiotics in an internal active site, where a heme group is bound to a specific cysteine residue inside a big hydrophobic cavity [11]. To evaluate the potential of potato CYP81 family members to interact with the most prominent FM-EO components, machine learning models, based on known CYP450 chemical reactions, and in silico blind docking simulations were used to estimate spatial orientation and fit. A biologically significant favorable chemical and binding interaction was predicted between potato CYP81s and FM-EO constituents. The majority of predicted potato CYP81 proteins contained a heme-binding domain, and a large subset of them, after aligning them to known CYP450 structures from plants, had the heme group and the cysteine residue in a favorable position inside the large internal pocket, as well as the substrate positioned and oriented in a favorable manner, suggesting a correct heme group assembly and a potential for catalytic interaction. The compound from FM-EO most likely to interact with potato CYP81 family members was (*Z*)-β-ocimene, a highly volatile acyclic monoterpene. Interestingly, this compound was not the most abundant constituent of FM-EO [30], but exhibited strong interaction potential with the most DE *CYP81s* in potato exposed to FM-EO (*81D1-1* and *81D11-1*). Given the large size and potential flexibility of the cavity [62], other hydrophobic molecules of similar size could also serve as substrates, which is in line with the nature of the CYP81 family in other plants [17]. These in silico methods attempt to predict biological patterns and capture the evolutionary logic of CYP450–substrate binding to determine whether the reaction is chemically and geometrically possible, but they are in no way scientifically conclusive. More detailed research and in vitro tests are needed to determine actual CYP450–substrate binding in potato.

Apparently, the substrate-promiscuous nature of some CYP450 members could be central for detoxification pathways in plants, allowing them to process a wider array of metabolites using a limited set of enzymes. Indeed, there are examples of CYP450s that metabolize terpenes that also metabolize volatile and hydrophobic herbicides [11], while others that metabolize phenylpropanoids (CYP73A1) or fatty acids (CYP81B1) also metabolize herbicides to some extent [14]. On the other hand, due to their inherent ability to synthesize secondary metabolites, plants contain a much larger subset of potential xenobiotic biotransforming enzymes than animals, some possibly specialized for detoxification, while others, normally involved in endogenous biosynthesis, could be mistaking xenobiotics for plants’ secondary metabolites [4]. It has been suggested before that some naturally synthesized plant metabolites, such as phenylpropanoids, are recognized and metabolized in receiving plants using the same enzymatic systems (CYP450s and GSTs) as synthetic herbicides and other xenobiotics [74]. Whatever the underlying evolutionary reason—the need to detoxify a large array of xenobiotics, or the diversification of plant secondary metabolic pathways—plants seem to be vastly adept at dealing with allelochemicals and various toxins. Uptake of VOCs presents recipient plants with an apparent physiological trade-off. Volatile molecules must persist long enough to activate defense signaling, yet excessive intracellular accumulation can disrupt membrane integrity or redox homeostasis. Consequently, rapid CYP450-mediated transformation may simultaneously terminate the chemical signal and prevent toxicity, while generating metabolites that remain biologically active or become substrates for downstream conjugation pathways.

## 5. Conclusions

Our findings suggest that the uptake of volatiles from FM-EO triggers a coordinated metabolic response in potato. Given that FM-EO is dominated by highly reactive terpenoids, the pronounced induction of CYP450 genes points to CYP-mediated oxidation as a key initial step determining the metabolic fate of absorbed VOCs. Oxidative conversion of hydrophobic terpenes is likely to generate more polar intermediates that subsequently become suitable substrates for glutathione S-transferase (GST)-mediated conjugation. Notably, *CYP81* genes exhibited a clear induction pattern, identifying this subfamily as a promising candidate for mediating the potato response to volatile exposure.

Comprehensive bioinformatic analyses characterized the inducible *81D1-1*, which showed the highest expression among all *CYP81* genes in the microarray analysis, as a CYP450 containing a predicted N-terminal hydrophobic signal or membrane-anchor region, with the conserved P450 heme-binding signature, and predicted transcription factor binding sites associated with oxidative stress responses. Interestingly, *81D11-1*, for which bioinformatic prediction provided only limited functional annotation, identifying it solely as a heme-binding protein, displayed the second-highest expression level. Nevertheless, the presence of numerous predicted cis-regulatory elements within its promoter region (seven distinct types) suggests a high degree of transcriptional plasticity and potential involvement in VOC metabolism. Molecular docking further revealed favorable binding affinities of both enzymes toward the volatile constituents of FM-EO, collectively identifying these two CYP81 enzymes as strong candidates for the oxidative metabolism of absorbed volatile terpenes in potato.

Together with the previously observed depletion of reduced glutathione and the induction of *GST* transcripts, these findings support a putative sequential detoxification mechanism involving CYP81-dependent oxidation followed by glutathione conjugation. We propose that this coordinated metabolic response enables potato plants to detoxify, sequester, and compartmentalize absorbed volatile terpenes while maintaining cellular redox homeostasis during volatile-mediated plant–plant interactions.

Future studies employing gene silencing will be required to verify the functional role of these *CYP81* genes in VOC metabolism in receiver plant cells. In addition, identifying and localizing GST–volatile conjugates within cellular compartments will be essential for validating the proposed detoxification pathway and elucidating the intracellular fate of absorbed VOCs. Beyond advancing our understanding of volatile terpene metabolism, elucidation of the CYP81-dependent detoxification pathway may facilitate the rational application of EOs as biopesticides by maximizing their efficacy while minimizing phytotoxic effects.

## Figures and Tables

**Table 1:** The top eight differentially expressed (DE) CYP450 unique transcripts, defined by NCBI Gene Description, Primary Accession Nucleotide, and Primary Accession Protein categories, with the fold change (fc) values in cDNA microarray analysis.

Short Label	Cytochrome P450 Gene Description	Primary Accession Nucleotide	Primary Accession Protein	fc
*CYP72-1*	CYP72A219-like	XM_015309158.1	XP_015164644.1	31.95
*CYP72-2*	CYP72A219-like	XM_006348243.2	XP_006348305.1	30.39
*CYP72-* *3*	CYP72A219-like	XM_006348244.2	XP_006348306.1	28.51
*81D1-1*	81D1-like	XM_006339781.1	XP_006339843.1	27.95
*CYP72-* *4*	CYP72A219-like	XM_006348242.2	XP_006348304.1	27.23
*81D11-1*	81D11-like	XM_015305409.1	XP_015160895.1	26.93
*81D1-2*	81D1-like	XM_006362014.2	XP_006362076.1	12.68
*CYP72-* *5*	CYP72A219-like	XM_006348238.2	XP_006348300.2	9.38

**Table 2:** Classification and number (No.) of potato DE cDNA microarray transcripts in CYP450 subfamilies.

CYP450 Subfamily	E-Class I (No.)	E-Class IV (No.)	Unclassified (No.)
CYP72A219-like	14		
CYP736A12-like	5		
83B1-like	5		
71A3-like	3		
71A4-like	2		1
98A2-like	2		
704C1-like	2		
76A2-like	2		
CYP82D47-like	2		
77A1	2		
81D1-like	2		
90B1		1	
71A1-like	1		
82C4-like	1		
71A9-like	1		
734A1-like	1		
94A2-like	1		
84A1	1		
71A25-like	1		
85A1	1		
81D11-like	1		
86A22	1		
86A8-like	1		
Total	52	1	1

**Table 3:** CDD classes of potato DE CYP450s.

CDD Classes	No.
cd11072—CYP71-like	15
cd20642—CYP72	11
cd11064—CYP86A	5
cd11043—CYP90-like	2
cd20653—CYP81	2
cd20654—CYP82	2
cd11073—CYP76-like	2
cd11075—CYP77_89	2
cd20656—CYP98	1
Total	42

**Table 4:** Representative domains of potato DE CYP450s detected by InterPro.

Cytochrome P450 Representative Domains	No.
Phobius—signal peptide region (SP)	24
Phobius—predicted transmembrane region (TM)	31
Phobius—predicted extracellular region (EC)	49
Phobius—predicted cytoplasmic region (CP)	31
TMHMM—transmembrane helix prediction	35
ProSitePatterns (PS00086)—CYP450 cysteine heme–iron ligand signature	44

**Table 5:** InterPro characterization of the top eight most DE potato CYP450s by representative protein structure domains.

Short Label	SP	TM	EC	CP	TMHMM	PS00086
*CYP72-1*		+	+	+		
*CYP72-2*		+	+	+		
*CYP72-* *3*		+	+	+		
*81D1-1*	+		+		+	+
*CYP72-* *4*		+	+	+	+	
*81D11-1*						+
*81D1-2*		+	+	+	+	+
*CYP72-* *5*	+	+	+	+	+	

SP—signal peptide region; TM—transmembrane region; EC—extracellular region; CP—cytoplasmic region; TMHMM—transmembrane helix prediction; PS00086—cysteine heme–iron ligand signature.

## Data Availability

Supporting data can be found as a supplement to this paper, and in the RADaR database—Digital Repository of Archived Publications Institute for Biological Research “Siniša Stanković” at: https://radar.ibiss.bg.ac.rs/handle/123456789/8350 (accessed on 29 August 2026) and https://radar.ibiss.bg.ac.rs/handle/123456789/8179 (accessed on 29 August 2026).

## Supplementary Materials

The following supporting information can be downloaded at: https://www.mdpi.com/article/10.3390/antiox15091107/s1, Figure S1: Protein–protein interaction network of potato CYP81 proteins. Nodes represent proteins, while lines represent different types of interactions; Figure S2. GO enrichment analysis of protein–protein interaction network of potato CYP81 proteins. Table S1: Potato CYP450 gene primers used RT-qPCR analyses; Table S2: CYP450 transcripts detected by cDNA microarray in potato plants exposed to French marigold for 8 h; Table S3: CYP450 genes of potato family CYP81 identified in the whole potato genome using the Spud DB Potato Genomics Resource, v6.1; Table S4: InterPro classification and domain information of CYP81 protein family of potato; Table S5: Physical and chemical properties of potato CYP81 proteins; Table S6: Top 20 putative cis-acting regulatory elements identified in the 5′regulatory region in *CYP81* potato genes, predicted by PlantCARE; Table S7: Potato CYP81 proteins mapped to related Arabidopsis proteins by the STRING database; Table S8: The closest protein–protein interactions of mapped genes obtained from the STRING database; Table S9: Cavity-detection guided blind docking of potato CYP81 proteins with FM-EO components, predicted by CB-Dock2.

## Abbreviations

The following abbreviations are used in this manuscript:

VOC	volatile organic compound
CYP450	cytochrome P450 enzyme
*GST*	glutathione-S-transferase
ROS	reactive oxygen species
FM-EO	French marigold (*Tagetes patula* L.) essential oil
qPCR	quantitative real-time PCR
fc	fold change in cDNA analysis
FC	fold change in qPCR analysis
log_2_fc	log_2_-transformed fold changes in cDNA analysis
log_2_FC	log_2_-transformed fold changes in qPCR analysis
LSD	Fisher’s least significant difference test
EDTA	ethylenediaminetetraacetic acid
DTT	dithiothreitol
PVPP	polyvinylpolypyrrolidone
BSA	bovine serum albumin
SDS-PAGE	sodium dodecyl sulfate–polyacrylamide gel electrophoresis
PBS	phosphate-buffered saline
CJ	cis-jasmone
MeJA	methyl jasmonate
pI	isoelectric points
chr	chromosome
CP	cytoplasmic
TM	transmembrane
TMHMM	transmembrane helix prediction
EC	extracellular
CDD	Conserved Domain Database
DE	differentially expressed
Cys	cysteine
SG	the gamma-sulfur
BFVD	Big Fantastic Virus Database
AFDB50	AlphaFold Database clustered to 50% sequence identity
ATBC	AllTheBacteria
ESP	enzyme-substrate prediction
DOG	Domain Graph package
pLDDT	predicted local distance difference test scores
3D	three-dimensional
TF	transcription factor
CRE	cis-regulatory element
RADaR	Digital Repository of Archived Publications Institute for Biological Research “Siniša Stanković”
GO	Gene Ontology
GSDS	Gene Structure Display Server 2.0 software
UTR	untranslated region
CDS	coding DNA sequence
